# Evolutionary Origin and Diversification of Epidermal Barrier Proteins in Amniotes

**DOI:** 10.1093/molbev/msu251

**Published:** 2014-08-27

**Authors:** Bettina Strasser, Veronika Mlitz, Marcela Hermann, Robert H. Rice, Richard A. Eigenheer, Lorenzo Alibardi, Erwin Tschachler, Leopold Eckhart

**Affiliations:** ^1^Research Division of Biology and Pathobiology of the Skin, Department of Dermatology, Medical University of Vienna, Vienna, Austria; ^2^Department of Medical Biochemistry, Medical University of Vienna, Vienna, Austria; ^3^Department of Environmental Toxicology and Forensic Science Graduate Program, University of California–Davis; ^4^Proteomics Core Facility, Genome Center, University of California–Davis; ^5^Dipartimento di Scienze Biologiche, Geologiche ed Ambientali (BiGeA), University of Bologna, Bologna, Italy

**Keywords:** epidermis, reptiles, birds, gene family, gene fusion

## Abstract

The evolution of amniotes has involved major molecular innovations in the epidermis. In particular, distinct structural proteins that undergo covalent cross-linking during cornification of keratinocytes facilitate the formation of mechanically resilient superficial cell layers and help to limit water loss to the environment. Special modes of cornification generate amniote-specific skin appendages such as claws, feathers, and hair. In mammals, many protein substrates of cornification are encoded by a cluster of genes, termed the epidermal differentiation complex (EDC). To provide a basis for hypotheses about the evolution of cornification proteins, we screened for homologs of the EDC in non-mammalian vertebrates. By comparative genomics, *de novo* gene prediction and gene expression analyses, we show that, in contrast to fish and amphibians, the chicken and the green anole lizard have EDC homologs comprising genes that are specifically expressed in the epidermis and in skin appendages. Our data suggest that an important component of the cornified protein envelope of mammalian keratinocytes, that is, loricrin, has originated in a common ancestor of modern amniotes, perhaps during the acquisition of a fully terrestrial lifestyle. Moreover, we provide evidence that the sauropsid-specific beta-keratins have evolved as a subclass of EDC genes. Based on the comprehensive characterization of the arrangement, exon–intron structures and conserved sequence elements of EDC genes, we propose new scenarios for the evolutionary origin of epidermal barrier proteins via fusion of neighboring S100A and peptidoglycan recognition protein genes, subsequent loss of exons and highly divergent sequence evolution.

## Introduction

Adaptations of the epidermis played key roles in the evolution of vertebrates that colonized the land in the lower Carboniferous (Chuong et al. 2002). In particular, the evolution of an efficient protection against cutaneous water loss was a crucial event in the transition of amniotes to a fully terrestrial lifestyle (Alibardi 2003; Maderson 2003; Madison 2003). Later, evolutionary innovations such as hair, mammary glands, and feathers, all of which represent modifications of the epidermis with contributions of the underlying mesenchyme (Wu et al. 2004), were the defining events in the appearance of mammals and birds, respectively.

The main cell type present in the epidermis, the keratinocyte, forms both the cornified layer (stratum corneum) and the mechanically resilient components of skin appendages. Differentiation of keratinocytes in the epidermis, hair, and claws/nails culminates in cornification, a mode of programmed cell death that involves the covalent cross-linking of structural proteins via transglutamination of lysine and glutamine residues (Candi et al. 2005; Alibardi 2006; Eckhart et al. 2013). Ultimately, so-called corneocytes, consisting of keratin filaments and a cross-linked protein envelope with covalently attached lipids, are formed. The most abundant proteins in the mammalian cornified envelope are involucrin, small proline-rich proteins (SPRRs), S100A proteins and loricrin, with the latter reportedly making up approximately 70% of total proteins (Rice and Green 1977, 1979; Steinert and Marekov 1995; Robinson et al. 1997; Kalinin et al. 2002).

Many structural proteins of mammalian corneocytes are encoded by genes that are clustered in a single chromosomal locus, the so-called epidermal differentiation complex (EDC) (Mischke et al. 1996; Kalinin et al. 2002). Genes of the S100A family are localized at the borders of the EDC whereas the central region of the EDC is occupied by loricin, involucrin and SPRR genes as well as by the genes coding for the families of late cornified envelope (LCE) proteins and the S100-fused type proteins (SFTPs) such as filaggrin (FLG) (Henry et al. 2012). In addition, the genes encoding the antimicrobial peptidoglycan recognition proteins (PGLYRPs) 3 and 4 (Kashyap et al. 2011) are localized in the EDC. Mutations in the *FLG* and LCE (*LCE3B*/*C*) genes are associated with the highly prevalent skin barrier diseases, atopic dermatitis and psoriasis, respectively (Palmer et al. 2006; de Cid et al. 2009). The EDC is conserved among mammals (Henry et al. 2012; Jiang et al. 2014).

The molecular organization of the epidermis of nonmammalian tetrapods including sauropsids, that is, reptiles and birds, has started to emerge in recent years. Type I and type II keratins (also known as alpha-keratins), including important cytoskeletal proteins of keratinocytes (Schweizer et al. 2006), have been identified in sauropsids (Vandebergh and Bossuyt 2012), and cysteine-rich keratins homologous to mammalian hair keratins were shown to be expressed in the claws of lizards (Eckhart et al. 2008). The epidermis and the skin appendages of reptiles and birds also contain beta-keratins, which are defined by the presence of a unique sequence domain that is different from the intermediate filament domain of true keratins (Gregg et al. 1984; Alibardi et al. 2009). Phylogenetic analyses of beta-keratins have suggested that they had a key role in the evolution of feathers (Dalla Valle et al. 2008; Greenwold and Sawyer 2010). In contrast to previous comparative histological investigations that were based on the cross-reactivities of antibodies raised against mammalian proteins (Hohl 1990; Alibardi 2006), recent studies of the sauropsidian epidermis have utilized newly available genome data and specific immunoreagents (Eckhart et al. 2008; Vanhoutteghem et al. 2008).

Here, we used a comparative genomics-based approach to screen for novel proteins of the epidermis in sauropsids. We identified homologs of the EDC in the chicken (*Gallus gallus*) and in the green anole lizard (*Anolis carolinensis*) and demonstrate that more than 20 previously uncharacterized genes within these regions are expressed in a keratinocyte-specific manner. Together with identification of conserved sequence motifs and the comparison of exon–intron structures of EDC genes, these data suggest a new scenario for the evolutionary origin of EDC genes and for their diversification during the evolution of amniotes.

## Results

### Comparative Genomics and *de novo* Gene Predictions Define the EDC in Chicken and Anole Lizard

**Fig. 1.**
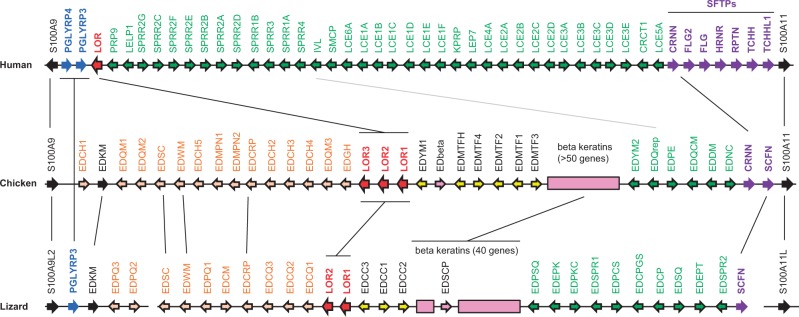
Organization of the EDC in sauropsids. Genes of the EDC in human (chromosome 1q21), chicken (chromosome 25), and green anole lizard (locus not yet assigned to a chromosome) are schematically depicted. Arrows indicate the orientation of the genes. SEDC genes with two exons are represented by colored arrows with a black frame whereas other genes are shown as filled arrows. Clusters of beta-keratin genes are shown as boxes. Colors indicate groups of genes as defined in the text. Black vertical lines connect orthologs; a gray line connects putative orthologs. Note that the schemes are not drawn to scale.

To establish a basis for the phylogenetic analysis of the EDC, we screened the genomes of nonmammalian vertebrates for the presence of loci homologous to the mammalian EDC. Besides performing BLAST searches with mammalian EDC genes as queries, we investigated the genomic regions flanking S100A genes and searched for genes with an exon–intron organization identical to that of human EDC genes. In the human EDC, genes of two main types of exon–intron organization are located between *S100A9* and *S100A11* (fig. 1). First, there are genes consisting of a 5′-terminal noncoding exon and a second exon comprising the entire coding region. We will refer to these genes as “simple EDC” (SEDC) genes and to the proteins encoded by them as SEDC proteins. The second class of genes, encoding SFTPs, consists of a 5′-terminal noncoding exon and two coding exons (Henry et al. 2012). *PGLYRP3* and *PGLYRP4* consist of seven and nine exons, respectively.

In the genomes of ray-finned fishes (*Takifugu rubripes* and others), the coelacanth (*Latimeria chalumnae*), and amphibians (*Xenopus tropicalis*, *X. laevis*) S100A genes are not flanked by genes homologous to SEDC, SFTP, or PGLYRP genes (data not shown). By contrast, genomic regions comprising *S100A9* and *S100A11* as well as SEDC and SFTP genes were identified in the chicken and the green anole lizard (fig. 1). The in-depth characterization of sauropsidian SFTPs was reported elsewhere (Mlitz et al. 2014). SEDC genes of the chicken and the lizard were identified by BLAST searches and by *de novo* gene predictions in the region flanked by *S100A9* and *S100A11*. The latter approach involved the careful screening of genomic DNA sequences for open reading frames that 1) are preceded by a splice acceptor site (pyrimidine tract, and AG) at a typical distance of 20–25 nt upstream of the start codon and 2) encode proteins similar to mammalian SEDCs either with regard to high contents of the amino acids, cysteine, proline, glycine, serine, or glutamine, and/or with regard to the presence of distinct sequence motifs defined for mammalian EDC proteins (Backendorf and Hohl 1992). The screening was done as an iterative process in which the amino acid sequences of newly identified SEDC proteins were used as queries for BLAST searches. This facilitated the identification of additional hits in the genomes of the chicken and lizard whereas no SEDCs were found in fish and amphibians including the Chinese salamander (*Hynobius chinensis*) for which a transcriptome of the whole body has been published recently (Che et al. 2014).

**Fig. 2.**
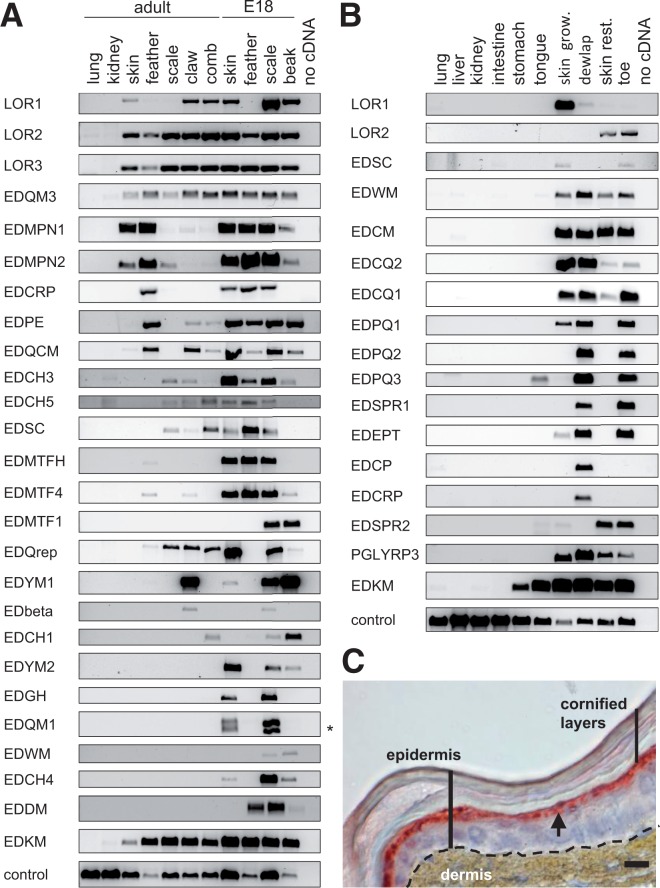
EDC genes of sauropsids are differentially expressed in the skin of different body sites and in other tissues that contain keratinocytes. The expression of EDC genes was determined by RT-PCR in tissues of the chicken (*A*) and of the green anole lizard (*B*). Images of RT-PCR products are ordered to highlight similarities of expression patterns of individual genes. Amplification of EDQM1 cDNA yielded two PCR products (asterisk) that result from two alleles of this gene. Note that the RT-PCR screening of these tissue panels (*A* and *B*) was performed on a subset of the predicted EDC genes. The skin of the green anole lizard was immunostained (red) with an antibody against lizard loricrin (*C*). The arrow points to the suprabasal epidermal layer in which loricrin is expressed. Skin grow., skin growing; skin rest., skin resting. Scale bar, 10 µm.

Subsequently, the noncoding exon 1 of SEDC genes was searched by screening of the upstream sequence for a bona fide splice donor motif (G-GTAAG) preceded by a TATA box at a distance of 60–90 nt. The initial in silico screening was complemented by rapid amplification of cDNA ends (RACE) to determine the 5′-terminal sequences of selected cDNAs (supplementary table S3, Supplementary Material online) which were aligned to the genomic sequence to map exon 1 of the corresponding genes. Iterative rounds of screening facilitated the definition of exon 1 candidates for 88% and 97% SEDC genes of lizard and chicken, respectively. To test the expression of these genes (supplementary tables S1 and S2 and fig. S1, Supplementary Material online), we designed intron-spanning primer pairs (supplementary table S4, Supplementary Material online) and performed reverse transcription (RT)-polymerase chain reactions (PCRs) on RNAs extracted from various tissues of chicken and lizard. Indeed, more than 60% of the SEDC predictions for the lizard and more than 90% of those for the chicken could be verified (supplementary table S2, Supplementary Material online and fig. 2).

We noted that beta-keratin genes conformed to the two-exon criterion for SEDCs and that these genes formed a subcluster within the cluster of SEDC genes of the chicken and the lizard (fig. 1), suggesting that beta-keratins represent a subtype of SEDCs. In addition to the previously characterized beta-keratins of the chicken (Greenwold and Sawyer 2010), we identified a beta-keratin-like protein (EDbeta) that was encoded by a gene localized outside of the main beta-keratin cluster of chicken chromosome 25 (fig. 1 and supplementary fig. S2, Supplementary Material online). In the lizard, the beta-keratin cluster (Dalla Valle et al. 2010) was interrupted by the SEDC gene, *EDSCP*, which lacked a beta-keratin core box (fig. 1 and supplementary fig. S2, Supplementary Material online).

In total 30 and 26 SEDC genes, in addition to those encoding 51 and 40 beta-keratins (Dalla Valle et al. 2010; Greenwold and Sawyer 2013) were identified in chicken and lizard, respectively (fig. 1). This compares to 37 SEDC genes in the human genome (fig. 1). Two genes of the lizard and three genes of the chicken were orthologous to human loricrin, reportedly the most abundant component of the protein envelope of cornified keratinocytes (Mehrel et al. 1990) and hence were also named loricrins (supplementary fig. S3, Supplementary Material online). Orthology was judged by criteria of reciprocal highest sequence similarity (Koonin 2005), gene locus synteny and conserved exon–intron structure. The other SEDC genes were not clearly orthologous to any particular human SEDC gene but showed equal similarity to several human SEDC genes. To indicate the likely involvement of these genes in epidermal differentiation and to highlight some distinctive features of the amino acid sequences encoded by them, we assigned tentative names to the sauropsidian SEDC genes. The gene names consist of a common part, that is “epidermal differentiation (ED) protein” and a specific part, such as “rich in cysteine (C) and histidine (H)” (abbreviation of the complete name, EDCH). For reasons of convenience, we will use abbreviated gene names and refer to supplementary table S1, Supplementary Material online, for the list of complete names.

In addition to the SFTP and SEDC genes, a peptidoglycan recognition receptor gene containing five exons was identified in the EDC of the green anole lizard in a position corresponding to those of its human homologs *PGLYRP3* and *PGLYRP4* (fig. 1). The EDC of the chicken lacked a PGLYRP gene. Moreover, we identified a gene named *EDKM* close to the *S100A9*-side end of the EDC in both chicken and lizard. *EDKM* genes contained four exons of which exons 2 and 3 contained an open reading frame that encodes a protein of weak similarity to S100A proteins (supplementary fig. S4, Supplementary Material online).

### Chicken and Anole Lizard EDC Genes Are Differentially Expressed in Tissues Containing Keratinocytes

To determine the expression pattern of the predicted genes, we performed RT-PCRs on RNAs derived from various tissues of chicken and green anole lizard. All EDC genes were expressed in at least one tissue that contained keratinocytes such as skin, skin appendages, or the tongue but not in tissues lacking keratinocytes such as lung and kidney (fig. 2 and supplementary fig. S5, Supplementary Material online).

The expression patterns varied considerably among the genes. In the chicken, loricrin homologs and *EDQM3* showed a relatively uniform expression in embryonic and adult skin and skin appendages whereas other SEDC genes were predominantly expressed in a subset of samples, for example, *EDYM1* was strongly expressed in the claws and in the beak but not in feathers whereas *EDMPN1*, *EDMPN2*, *EDCRP*, *EDPE*, and *EDQCM* were predominantly expressed in feathers (fig. 2*A*). Reanalysis of a proteomic data set from hard-cornified skin appendages of the chicken (Rice et al. 2013) demonstrated that proteins of 14 newly identified EDC genes were components of the beak, claws, feathers, and/or leg scales (supplementary fig. S7 and table S5, Supplementary Material online). The distribution of the peptide hits matched largely, but not completely, the distribution of the corresponding mRNAs as determined by RT-PCR (fig. 2*A*).

In the green anole lizard, loricrin 1 was predominantly expressed in skin samples containing epidermis in the renewal phase of the shedding cycle, whereas loricrin 2 was expressed at highest levels in the toes (fig. 2*B*). Interestingly, many genes were expressed in the dewlap and in toe but not in abdominal skin, possibly in correlation with the amount of interscale epidermis that facilitates flexible extensions of skin in moving body parts (fig. 2*B*). *PGLYRP3* was expressed specifically in samples containing epidermis but not in those from internal organs. Like its ortholog in the chicken (fig. 2*A*), *EDKM* of the lizard showed the broadest expression pattern of all EDC genes which included the stomach and the tongue as well as all the epidermal samples (fig. 2*B*). Collectively, these RT-PCR data demonstrated differential expression of EDC genes in different skin sites of the two species of sauropsids.

To determine the expression pattern of a representative SEDC protein in situ, we generated an antibody against loricrin 1 of the lizard and performed an immunohistochemical analysis. Lizard loricrin 1 was specifically expressed in suprabasal epidermal keratinocytes underneath the cornified cell layers (fig. 2*C* and supplementary fig. S6, Supplementary Material online), thus resembling the distribution of loricrin in human epidermis (Maestrini et al. 1996). Of note, loricrin is present in cornified cells but its epitopes are masked due to cornification (Mehrel et al. 1990). In line with the RT-PCR results (fig. 2*B*), loricrin 1 was expressed in the growth phase of the epidermal shedding cycle (fig. 2*C*) but not or only weakly in the resting epidermis (data not shown).

### SEDC Genes Encode Proteins that Are Either Rich in Glycine and Serine, Cysteine and Proline or Glutamine

**Fig. 3.**
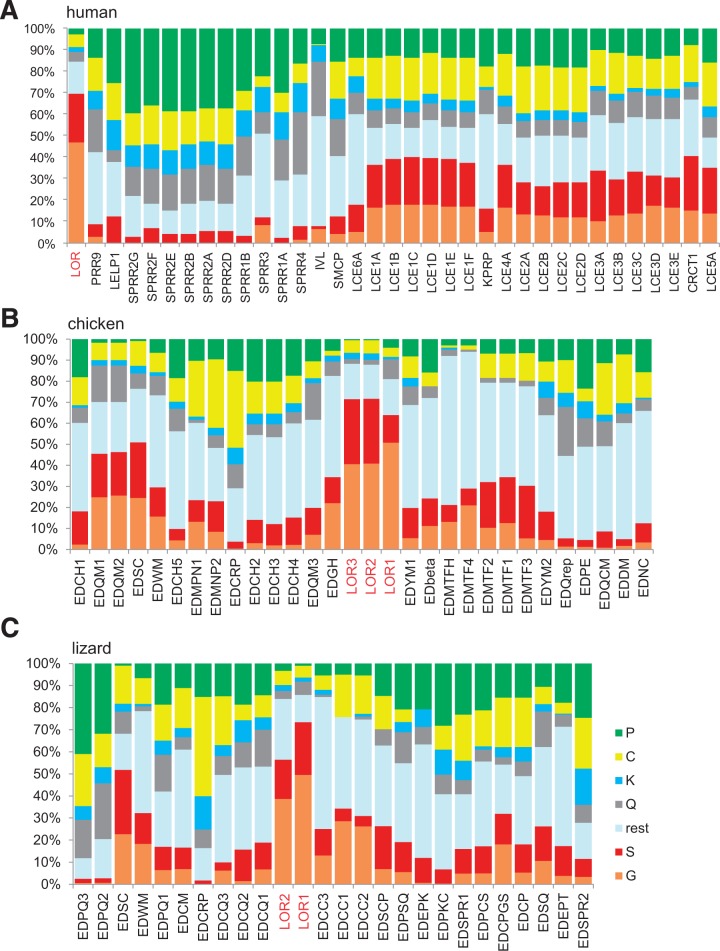
SEDC proteins have evolved highly diverse contents of amino acid residues in mammals and sauropsids. The diagrams show the amino acid compositions of SEDC proteins of human (*A*), chicken (*B*), and the green anole lizard (*C*). The protein data are shown in the order of the corresponding genes in the EDC (fig. 1). For better overview, the homologous loricrin proteins of the three species are highlighted with red letters.

Similar to the human SEDC proteins, the SEDC proteins of the chicken and the green anole lizard are enriched for a small subset of amino acid residues which, however, varies among individual SEDCs (fig. 3). High contents of proline were found in chicken EDPE (20% of total amino acids) and lizard EDPQ2, EDPQ3, and EDPKC. High contents of cysteine are present in EDCRP of the chicken (40% of total amino acid residues) and lizard (35%). Glycine and serine were particularly prominent in orthologs of mammalian loricrin (fig. 3). These two amino acids together accounted for approximately 70% of total amino acid residues in all three chicken loricrin homologs and in the two loricrin homologs found in the lizard. High glycine and serine contents were also found in EDSC of which orthologs are present in both chicken and lizard, as well as in EDQM1 and EDQM2 in the chicken. Potential target sites of transglutamination, that is, glutamine and lysine residues, are abundant in almost all SEDCs; however, lizard EDCC1 and EDSCP as well as chicken EDbeta and EDMTF1-4 lacked lysine. SEDC proteins encoded by neighboring genes within the EDC typically have similar amino acid compositions, indicating evolution of SEDCs by gene duplications and adjacent arrangement of daughter genes (fig. 3).

The SEDC proteins of chicken and lizard showed a considerable size distribution that was comparable to that of human SEDC proteins. Most sauropsidian SEDCs as well as all but three human SEDC proteins have an amino acid residue number between 70 and 170 (supplementary fig. S8, Supplementary Material online) which corresponds to a molecular weight range of 8–20 kDa. Three human SEDCs, that is, loricrin (312 amino acid residues), KPRP (579 amino acid residues), and involucrin (585 amino acid residues) are markedly longer than other human SEDCs. Likewise, loricrins (more than 500 amino acid residues) and several long SEDCs of chicken and lizard have more than 300 amino acid residues (supplementary fig. S8, Supplementary Material online). As these proteins contained repetitive sequence elements in their central region, their evolution is likely to have involved unequal crossover and selection for genes encoding longer proteins.

Of note, chicken EDQrep resembles human involucrin with regard to having a large number of glutamine (Q) residues and a highly repetitive sequence. A protein corresponding to the C-terminal portion of chicken EDQrep has recently been predicted and suggested to represent the chicken ortholog of mammalian involucrin because of these similarities (Vanhoutteghem et al. 2008) (supplementary fig. S9, Supplementary Material online). In contrast to the highly abundant Q residues, which dominate the amino acid sequence alignment of involucrin and EDQrep (supplementary fig. S9, Supplementary Material online), several other amino acid residues show very different abundance in these two proteins, for example, mammalian involucrins contain a maximum of seven cysteine residues (Phillips et al. 1997) whereas chicken EDQrep contains 76. Moreover, the isoelectric points (pI) of involucrin (pI 4.6) and EDQrep (pI 8.9) differ substantially.

### Distinct Amino Acid Sequence Motifs Are Conserved among Mammalian and Sauropsidian SEDC Proteins

**Fig. 4.**
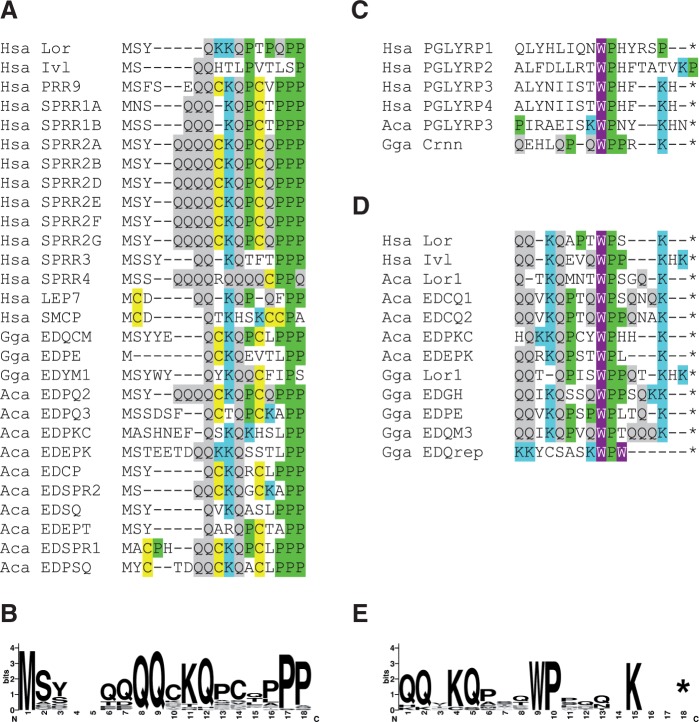
EDC proteins contain conserved amino acid sequence motifs at their amino-terminus and carboxy-terminus. (*A*) Amino acid sequence alignment showing the conserved sequence motif at the amino-terminus of SEDC proteins. (*B*) Sequence logo of the amino-terminal motif. (*C*) Chicken cornulin (Crnn) and PGLYRPs contain sequences similar to the conserved carboxy-terminal sequence motif of SEDCs (*D*). Note that the genes encoding PGLYRP1 and 2 are not located in the EDC. (*E*) Sequence logo of the carboxy-terminal motif of SEDCs. Amino acid residues involved in covalent molecular cross-linking (C, Q, K) as well as P and W are highlighted by color shading. Asterisks mark the end of the protein. Aca, *Anolis carolinensis*; Gga, *Gallus gallus*; Hsa, *Homo sapiens*.

Similar to human SEDC proteins, many sauropsidian SEDC proteins contain gene-specific repetitive sequence elements (supplementary fig. S10, Supplementary Material online). However, both the N-terminus and the C-terminus of several SEDC proteins contained conserved sequence motifs partly identical to the sequence domains defined for mammalian loricrin, involcrin, and SPRRs (Backendorf and Hohl 1992) (fig. 4). The N-terminal motif, MSYxxxxQQCKQPCQPPP (fig. 4*A* and *B*), represented a combination of potential target sites of transglutamination, that is, glutamine and lysine, cysteines that could serve in disulfide bridge formation and proline residues, which suppress the formation of the secondary protein structures, that is, alpha-helices and beta-sheets. This motif was found in human, chicken and lizard SEDCs (supplementary fig. S11, Supplementary Material online) indicating that it was inherited from an ancestral SEDC protein present in the last common ancestor of amniotes.

Another sequence motif, MCSRxxxxxCH is encoded specifically by SEDC genes located in the region between S100A9 and the loricrin homologs of the chicken and the lizard (supplementary figs. S11 and S12, Supplementary Material online). In contrast to the sauropsidian EDCs, mammalian EDCs lack SEDC genes between *S100A9* and *LOR*, indicating that the genes containing the above-mentioned motif have originated either in sauropsids after their divergence from mammals or that they have been lost in the mammalian lineage.

The C-terminus of some but not all SEDC proteins encoded in the human, chicken, and lizard genomes is formed by a conserved sequence motif with the consensus sequence QQxKQPSQWPxQxxK-stop (fig. 4*D* and *E*). This motif is also present in chicken cornulin (fig. 4*C*), which is an SFTP encoded by a gene with three exons (Mlitz et al. 2014). Moreover, the C-terminal portion of this motif is present in PGLYRPs encoded in the human EDC (PGLYRP3 and PGLYRP4) and in the lizard EDC (PGLYRP3) (fig. 4*C*). The chicken, which has been the only sauropsidian species included in a previously published phylogenetic analysis of PGLYRPs (Montaño et al. 2011) lacks a *PGLYRP* gene in the EDC, presumably due to the loss of this gene. Importantly, the core residues (WP) of the C-terminal SEDC motif are conserved in PGLYRP1 and PGLYRP2 (fig. 4*C*), which are phylogenetically older than PGLYRP3 and PGLYRP4 (Montaño et al. 2011) and whose genes are located outside of the EDC.

### A Scenario for the Origin and Diversification of the EDC

**Fig. 5.**
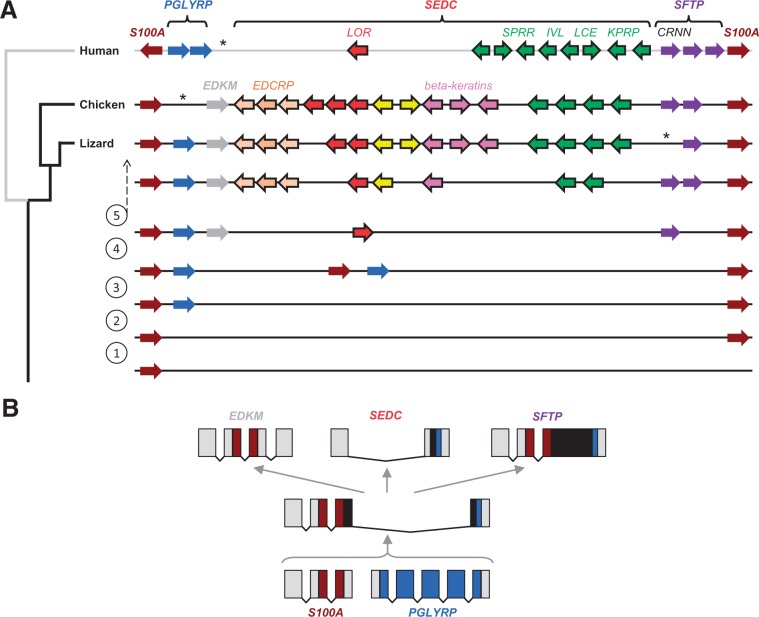
A scenario for the origin and diversification of EDC genes. (*A*) Data on the presence of conserved sequence elements as well as on the arrangement and orientation of genes in the EDC and their exon–intron structures were integrated into a hypothesis about the evolution of EDC genes. On the left, a phylogenetic tree leading to human, chicken, and green anole lizard is shown. The scheme on the right depicts the arrangement of genes in the EDC in these species as well as in their ancestors corresponding to the level of the phylogenetic tree. Asterisks indicate the positions of lost genes. To provide a better overview, only a subset of EDC genes of each clade (indicated by different colors) is shown. Encircled numbers refer to evolutionary steps that are described in the Results section. (*B*) Evolutionary origin of the distinct exon–intron organizations of EDC genes. One of several possible evolutionary pathways (supplementary fig. S13, Supplementary Material online) is depicted in simplified form. Exons are indicated by boxes, in which the noncoding regions are shaded gray and the coding regions are shaded in colors or in black. Identical colors indicate common ancestry and black indicates newly originated coding sequences. All genes shown in (*B*) are transcribed from left to right.

The conserved synteny of EDC gene loci, the distribution of conserved gene elements among EDC genes of mammals and sauropsids, and the application of the principle of parsimony, have led us to propose a comprehensive scenario for the evolution of the EDC. The hypothetical trajectory of gene innovation events is depicted in figure 5*A*. A scenario for the origin of the SEDCs, SFTPs, and EDKM is shown in figure 5*B*. Details of this scenario and of three alternative hypotheses on the origin of SEDCs and SFTPs are shown in supplementary figure S13, Supplementary Material online. The evolutionary scenario comprises the following main steps which are marked by encircled numbers in figure 5*A*:

1. An ancestral S100A gene was duplicated, perhaps multiple times, to give rise to a cluster of S100A genes (fig. 5*A*). Notably, the origin of *S100A1* and *S100A11*, which are located at the opposite ends of the EDC, dates back to early vertebrates (Zimmer et al. 2013).
2. An ancestral PGLYRP gene, located elsewhere in the genome, was duplicated and the copy, *PGLYRP3*, was inserted between S100A genes (fig. 5*A*). This insertion occurred before the divergence of the sauropsidian and mammalian lineages. Consequently, lizard *PGLYRP3* and human *PGLYRP3* and *PGLYRP4* are located at syntenic positions in the EDC (figs. 1 and 5*A*).
3. Additional duplications of S100A and PGLYRP genes (or parts of these genes) generated the precursors of SEDC and SFTP genes. These precursor genes were arranged in the same orientation of transcription which facilitated subsequent recombination events.
4. Adjacent S100A and PGLYRP genes of the primitive EDC underwent a series of fusions, duplications, loss of exons, loss of introns, and changes in the ends of their coding sequences, as depicted in supplementary figure S13, Supplementary Material online, leading to the origin of SEDC, SFTP, and EDKM genes. Figure 5*B* shows one of several possible examples of recombination routes that might have generated the distinct exon–intron structures of SEDCs, SFTPs, and EDKM as well as the distribution of conserved sequence elements, that is, the S100-like domains in S100A, SFTPs, and EDKM, and the C-terminal sequence motif in the last exons of PGLYRP3, many SEDCs, and at least one SFTP.
5. SEDCs and SFTPs underwent extensive rounds of gene duplication and sequence modifications that generated proteins of highly diverse sequences in which few sequence elements were conserved in a subset of proteins (fig. 4). EDC genes that are specific to sauropsids, such as beta-keratins, may have originated after the divergence of sauropsids from mammals or in a common ancestor of modern amniotes followed by gene loss in the mammalian lineage. Changes in the EDC gene composition contributed to the major adaptions of the integument to different terrestrial environments and lifestyles of sauropsids and mammals.

## Discussion

The results of this study demonstrate that not only mammals but also sauropsids have an EDC, implying that the EDC was already present in their last common ancestor. In fishes and amphibians, neither homologs of the EDC-specific genes of the SFTP or SEDC families nor any close arrangements of S100A genes and PGLYRP genes were found, indicating that the EDC originated after the divergence of amniotes from these clades of vertebrates. The future availability of additional amphibian genome sequences will help to better evaluate this point and to determine which precursors of the EDC were present in primitive amphibians. The currently available data suggest that the evolutionary origin of the EDC coincided with, and perhaps facilitated, the adaptation of a fully terrestrial lifestyle of amniotes.

The comprehensive characterization of EDC genes in phylogenetically diverse amniotes has allowed us to address the unresolved question about the evolutionary origin of EDC genes with different exon–intron organizations. The scenarios for the origin of SEDC and SFTP genes (fig. 5 and supplementary fig. S13, Supplementary Material online) integrate features of the gene arrangement in the EDC, the new finding of a conserved presence of a PGLYRP gene in the EDC of two different subclades of amniotes, the exon–intron structure of EDC genes and the distribution of common sequence elements among EDC proteins. Previously, Markova et al. (1993) have put forward the hypothesis that the fusion of an S100A gene to an EDC gene rich in sequence repeats (termed SEDC here) generated a gene encoding a protein with two distinct domains, that is, an S100 domain with calcium-binding activity and a repeat-rich domain interacting with the cytoskeleton. For the resulting genes, these authors coined the term “S100-fused genes” and the encoded proteins, such as filaggrin and trichohyalin, have been named SFTP since then. Our data indicate evolutionary pathways to the origin of SEDC genes and suggest that the original S100 fused gene hypothesis of Markova et al. (1993) is just one of several possible scenarios for the origin of SFTPs (supplementary fig. S13*B*, Supplementary Material online). We propose that the fusion of an S100A and a PGLYRP gene was the initial event in the evolution of both SEDC and SFTP genes. Subsequently, loss of exons and fusion of exons generated the different gene structures of SEDC and SFTPs, respectively (fig. 5*B* and supplementary fig. S13*A*, Supplementary Material online). Alternatively, the primordial SEDC gene might have inherited exon 2 and possibly also exon 1 from a PGLYRP precursor gene (supplementary fig. S13*B* and *C*, Supplementary Material online). In other scenarios, the similarity in the C-terminal sequence motifs of SEDCs, cornulin (an SFTP) and PGLYRPs (fig. 4*C* and *D*) has not been inherited from a common ancestral motif (supplementary fig. S13*A* and *B*, Supplementary Material online) but originated, by chance, more than once in different genes of the EDC (supplementary fig. S13*C* and *D*, Supplementary Material online). Although any model for the evolution of EDC genes must include speculative elements, a key role of gene duplications and fusions, as demonstrated for other cases of gene origin (Long et al. 2003; Chen et al. 2013) is supported by the results of the present study.

After the origin of the characteristic exon–intron structures in the ancestral SEDC and SFTP genes, the numbers of SEDC and SFTP genes expanded perhaps by the mechanism of gene duplication and subsequent mutation, as defined for other gene clusters (Chang and Duda 2012). Highly similar SEDC genes are mostly located in close vicinity, indicating that duplicated genes rarely underwent translocation but rather remained at the locus of their parent genes. In addition, gene conversion may have contributed to the evolution of sequence similarities between neighboring genes as well as to the evolution of sequence repeats within individual genes (Djian et al. 1993). To define the roles of purifying selection and concerted gene evolution (Nei and Rooney 2005) during the evolution of the EDC in different clades of sauropsids, including turtles, crocodilians, snakes, and tuatara, a careful analysis of EDC loci in newly sequenced genomes will be necessary in future studies. A comparison of our experimentally verified EDC genes with the gene annotations available in the GenBank at the time of completion of the present study (supplementary table S6, Supplementary Material online) suggests that manual curation and the investigation of gene expression, as performed in the present study, are instrumental in defining the EDC in new genomes.

Because this study has been designed to reveal the complete or near-complete set of EDC genes in chicken and the green anole lizard, it is now also possible to compare the number and sequence diversity of EDC genes in different clades of amniotes. Indeed, the EDCs of the chicken and the lizard comprise more genes than the human and other mammalian EDCs (Jiang et al. 2014) due to a higher number of SEDC genes which include the sauropsid-specific beta-keratin genes. It is possible that the number of SEDC genes correlates with the formation of particular features of the skin barrier to the environment or the ability to form clade-specific skin appendages.

Besides providing a framework for the study of the EDC, several of the results presented here have implications on the evolution of distinct components of the skin in amniotes. Our results of comparative genomics, RT-PCR screenings, immunohistochemical, and proteomics-based assays demonstrate that loricrin, previously reported to be the main component of the protein envelope of epidermal corneocytes (Mehrel et al. 1990), is also present in the epidermis of the chicken and the lizard. From these findings, it can be inferred that a loricrin gene has been present in the ancestral EDC of amniotes prior to divergence of mammals and sauropsids and that loricrin was a primordial component of the skin barrier of amniotes. Surprisingly, the anole lizard has two and the chicken has three homologs of loricrin. It remains to be determined whether the individual loricrin gene products have distinct physiological roles. Although the unique and extreme enrichment for glycine and serine residues is a distinctive feature of loricrin, the low sequence complexity of SEDC proteins makes it generally difficult to evaluate the orthology of individual SEDC proteins of mammals and sauropsids. Comparative analyses of closely related species within subclades of sauropsids will allow better definition of the phylogeny of SEDC genes.

Importantly, our data also suggest a hypothesis for the origin of beta-keratins, which have been the sole type of corneous proteins of sauropsids that has been characterized extensively in previous studies (Gregg et al. 1984; Dalla Valle et al. 2010; Greenwold and Sawyer 2010). We show that beta-keratins represent a subtype of SEDC proteins and hypothesize that the defining sequence motif of beta-keratins, that is, the so-called core box, evolved by mutations within an ancestral SEDC gene. Thus, the so-called beta-keratins are unrelated to keratins as defined by the human gene nomenclature committee (Schweizer et al. 2006). Here, we have still used the traditional term beta-keratins to conform with the literature; however, an alternative name such as corneous beta-proteins (Alibardi et al. 2009) should be considered.

The results of this study, together with previous reports on the evolution of beta-keratins (Dalla Valle et al. 2010; Greenwold and Sawyer 2010, 2011) and SFTPs (Mlitz et al. 2014), suggest a comprehensive scenario for the evolution of the EDC from two ancestral genes into a large cluster of genes with divergent expression patterns and highly divergent amino acid compositions of the encoded proteins. In particular, the evolution of SEDC genes is likely to have played an important role in the emergence of the different skin structures of amniotes. In future studies, the detailed characterization of individual SEDCs will provide new insights into the molecular architecture and evolution of scales, feathers, beak, and other sauropsidian skin appendages.

## Materials and Methods

### Sequence Queries and Alignments

DNA sequences of chicken (*G. gallus*), green anole lizard (*A. carolinensis*), and human (*Homo sapiens*) were obtained from GenBank (http://www.ncbi.nlm.nih.gov/, last accessed September 5, 2014). The tBLASTn algorithm and *de novo* gene predictions by “manual” screening for open reading frames and splice sites as well as amino acid sequence motifs in conceptually translated genome sequences were used to identify genes in the lizard and the chicken. Additional BLAST searches and investigations of the chromosomal loci flanked by at least one S100A gene were performed for fugu (*T. rubripes*), coelacanth (*L. chalumnae*), clawed frogs (*X. tropicalis*, *X. laevis*), Chinese salamander (*H. chinensis*) (whole-body transcriptome), platypus (*Ornithorhynchus anatinus*), opossum (*Monodelphis domestica*), sheep (*Ovis aries*), and mouse (*Mus musculus*). Amino acid sequences were aligned using various programs including Multalin (Corpet 1988). Sequence logos were generated using Weblogo (Crooks et al. 2004).

### Nucleic Acid Preparation, RT-PCR, and Sequence Analysis

Chicken and lizard genomic DNAs were prepared according to a standard protocol (Strauss 2001). RNA was extracted from homogenized tissues using peqGOLD TriFast (peqLab, Erlangen, Germany) and reverse transcribed with iScript cDNA Synthesis Kit (Bio Rad, Hercules, CA) according to the manufacturers’ instructions. gDNA and cDNA were amplified with DreamTaq DNA Polymerase (Thermo Scientific, Waltham, MA). Caspase-3 was amplified as control gene (Eckhart et al. 2008). Primer sequences are listed in supplementary table S4, Supplementary Material online. PCR products were purified and sequenced. The resulting sequences were submitted to the GenBank. The accession numbers referring to these sequences are listed in supplementary table S2, Supplementary Material online.

### Rapid Amplification of cDNA Ends

The 5′-ends of the lizard loricrin and EDCRP mRNAs were determined using the 5′/3′ RACE kit (Roche Applied Science, Basel, Switzerland) according to the manufacturer’s protocol. Sequences of the SP1 primers for gene-specific reverse transcription as well as sequences of SP2 and SP3 primers for two rounds of gene-specific nested PCRs are listed in supplementary table S3, Supplementary Material online.

### Proteomics

The data of a published proteomic analysis of chicken cornification products (Rice et al. 2013) were reanalyzed for peptides corresponding to chicken EDC proteins predicted in the present study. Protein identification criteria were the same as described previously (Rice et al. 2013). Only those proteins that could be detected at least two times in either the soluble or insoluble fraction of at least one tissue category (beak, claw, feather, or scale) were considered.

### Generation of an Anti-lizard Loricrin 1 Antiserum and Immunohistochemical Analysis

The lizard loricrin 1 antiserum was generated by immunizations of mice with the synthetic oligopeptide CLSQTKQMNTWPSGQK (corresponding to amino acid residues 720–735 of lizard locirin 1) (Genecust Europe, Dudelange, Luxembourg) coupled to keyhole limpet hemocyanin according to a published immunization protocol (Eckhart et al. 2008).

For immunohistochemical analysis, tissue samples were embedded in optimal cutting temperature compound, cryo-sectioned and fixed with acetone. Endogenous peroxidase was quenched by preincubation with 0.3% H_2_O_2_ in phosphate buffered saline (PBS). Anti-lizard loricrin 1 antiserum was used at a dilution of 1:1,000. As secondary antibody, biotinylated sheep anti-mouse immunoglobulin (1:200; GE, Chalfont, UK) was used together with 10% sheep serum to block unspecific binding. Specific red staining was obtained with streptavidin–biotin-horseradish peroxidase complex and chromogen 3-amino-9-ethylcarbazole (DakoCytomation, Glostrup, Denmark). The sections were counterstained with hematoxylin (Eckhart et al. 2008). To confirm the specificity of the staining, antisera preabsorbed with the immunization peptide (4 µg peptide per 1 µl antiserum) were used as a negative control. In other negative control experiments, preimmune serum was used instead of the anti-lizard loricrin 1 antiserum.

## Supplementary Material

Supplementary tables S1–S6 and figures S1–S13 are available at *Molecular Biology and Evolution* online (http://www.mbe.oxfordjournals.org/).

Supplementary Data
