# Supplementary material for: Evolutionary Origin and Diversification of Epidermal Barrier Proteins in Amniotes
Source: Mol Biol Evol. 2014 Aug 27;31(12):3194–205. doi: 10.1093/molbev/msu251 (PMC4245816; doi:10.1093/molbev/msu251)
Supplement: Supplementary Data [file supp_31_12_3194__index.html]

Evolutionary Origin and Diversification of Epidermal Barrier Proteins in Amniotes — Evolutionary Origin and Diversification of Epidermal Barrier Proteins in Amniotes — Supplementary Data 

# Evolutionary Origin and Diversification of Epidermal Barrier Proteins in Amniotes

## Supplementary Data

files

**Files in this Data Supplement:**

- Supplementary Data - pdf file
