# Supplementary material for: Evolutionary Origin and Diversification of Epidermal Barrier Proteins in Amniotes
Source: Mol Biol Evol. 2014 Aug 27;31(12):3194–205. doi: 10.1093/molbev/msu251 (PMC4245816; doi:10.1093/molbev/msu251)
Supplement: Supplementary Data [file supp_msu251_Strasser-et-at-Supplements.pdf]

Supplementary Data

Evolutionary origin and diversification of epidermal barrier proteins in amniotes

Bettina Strasser, Veronika Mlitz, Marcela Hermann, Robert H Rice, Richard A Eigenheer, Lorenzo Alibardi, Erwin Tschachler, Leopold Eckhart

Content

Supplementary Tables S1 – S6

Supplementary Figures S1 – S13

Suppl. Table S1

Tentative abbreviations and full names of SEDC and EDKM genes in the green anole lizard and the chicken

Species	Gene name abbreviation	Full gene name
Aca	*EDCC1*	Epidermal Differentiation protein containing Cysteine Cysteine motifs 1
Aca	*EDCC2*	Epidermal Differentiation protein containing Cysteine Cysteine motifs 2
Aca	*EDCC3*	Epidermal Differentiation protein containing Cysteine Cysteine motifs 3
Aca	*EDCM*	Epidermal Differentiation protein containing a CCCC Motif
Aca	*EDCP*	Epidermal Differentiation protein rich in Cysteine and Proline
Aca	*EDCPGS*	Epidermal Differentiation protein rich in Cysteine, Proline, Glycine and Serine
Aca	*EDCQ1*	Epidermal Differentiation protein rich in Cysteine and glutamine (Q) 1
Aca	*EDCQ2*	Epidermal Differentiation protein rich in Cysteine and glutamine (Q) 2
Aca	*EDCQ3*	Epidermal Differentiation protein rich in Cysteine and glutamine (Q) 3
Aca	*EDCRP*	Epidermal Differentiation Cysteine-Rich Protein
Aca	*EDEPK*	Epidermal Differentiation protein rich in glutamic acid (E), Proline and Lysine
Aca	*EDEPT*	Epidermal Differentiation protein rich in glutamic acid (E), Proline and Threonine
Aca	*EDKM*	Epidermal Differentiation protein containing a KKLIQQ Motif
Aca	*EDPCS*	Epidermal Differentiation protein rich in Proline, Cysteine and Serine
Aca	*EDPKC*	Epidermal Differentiation protein rich in Proline, lysine (K) and Cysteine
Aca	*EDPQ1*	Epidermal Differentiation protein rich in Proline and glutamine (Q) 1
Aca	*EDPQ2*	Epidermal Differentiation protein rich in Proline and glutamine (Q) 2
Aca	*EDPQ3*	Epidermal Differentiation protein rich in Proline and glutamine (Q) 3
Aca	*EDPSQ*	Epidermal Differentiation protein rich in Proline, Serine and glutamine (Q)
Aca	*EDSC*	Epidermal Differentiation protein rich in Serine and Cysteine
Aca	*EDSCP*	Epidermal Differentiation protein rich in Serine, Cysteine and Proline
Aca	*EDSPR1*	Epidermal Differentiation protein Small Proline Rich 1
Aca	*EDSPR2*	Epidermal Differentiation protein Small Proline Rich 2
Aca	*EDSQ*	Epidermal Differentiation protein rich in Serine and glutamine (Q)
Aca	*EDWM*	Epidermal Differentiation protein containing WYDP Motif
Aca	*LOR1*	Loricrin 1
Aca	*LOR2*	Loricrin 2
Gga	*EDbeta*	Epidermal Differentiation protein beta
Gga	*EDCH1*	Epidermal Differentiation protein containing Cysteine Histidine motifs 1
Gga	*EDCH2*	Epidermal Differentiation protein containing Cysteine Histidine motifs 2
Gga	*EDCH3*	Epidermal Differentiation protein containing Cysteine Histidine motifs 3
Gga	*EDCH4*	Epidermal Differentiation protein containing Cysteine Histidine motifs 4
Gga	*EDCH5*	Epidermal Differentiation protein containing Cysteine Histidine motifs 5
Gga	*EDCRP*	Epidermal Differentiation Cysteine-Rich Protein
Gga	*EDDM*	Epidermal Differentiation protein containing DPCC Motifs
Gga	*EDGH*	Epidermal Differentiation protein rich in Glycine and Histidine
Gga	*EDKM*	Epidermal Differentiation protein containing a KKLIQQ Motif
Gga	*EDMPN1*	Epidermal Differentiation protein containing a MPN sequence motif 1
Gga	*EDMPN2*	Epidermal Differentiation protein containing a MPN sequence motif 2
Gga	*EDMTF1*	Epidermal Differentiation protein starting with MTF motif 1
Gga	*EDMTF2*	Epidermal Differentiation protein starting with MTF motif 2
Gga	*EDMTF3*	Epidermal Differentiation protein starting with MTF motif 3
Gga	*EDMTF4*	Epidermal Differentiation protein starting with MTF motif 4
Gga	*EDMTFH*	Epidermal Differentiation protein starting with MTF motif and rich in Histidine
Gga	*EDNC*	Epidermal Differentiation protein encoded by Neighbor of Cornulin
Gga	*EDPE*	Epidermal Differentiation protein rich in Proline and glutamic acid (E)
Gga	*EDQCM*	Epidermal Differentiation protein containing QC Motifs
Gga	*EDQM1*	Epidermal Differentiation protein containing a glutamine (Q) Motif 1
Gga	*EDQM2*	Epidermal Differentiation protein containing a glutamine (Q) Motif 2
Gga	*EDQM3*	Epidermal Differentiation protein containing a glutamine (Q) Motif 3
Gga	*EDQrep*	Epidermal Differentiation protein containing glutamine (Q) repeats
Gga	*EDSC*	Epidermal Differentiation protein rich in Serine and Cysteine
Gga	*EDWM*	Epidermal Differentiation protein containing WYDP Motif
Gga	*EDYM1*	Epidermal Differentiation protein containing Y Motif 1
Gga	*EDYM2*	Epidermal Differentiation protein containing Y Motif 2
Gga	*LOR1*	Loricrin 1
Gga	*LOR2*	Loricrin 2
Gga	*LOR3*	Loricrin 3

NOTE - Aca, *Anolis carolinensis* ; Gga, *Gallus gallus.*

Suppl. Table S2A
Chicken EDC genes comprising 2 exons (SEDC genes)

	Gene predictions based on *Gallus gallus* genome sequence							Experimental data and protein predictions for *Gallus gallus* Tetra SL				
**Gene**	**Orientation of gene within EDC**	**Accession number of genomic DNA sequence**	**Start of coding sequence in exon 1**	**Exon 1 end**	**Exon 2 start**	**Start of coding sequence in exon 2**	**End of coding sequence**	**Expression confirmed by RT-PCR**	**Accession number of sequence submitted to GenBank**	**Source of sequence submitted to GenBank**	**Differences in encoded proteins (relative to prediction from reference sequence)**	**Length of coding sequence (nucl.)**
EDCH1	+	NC_006112.2	n.a.	743641	744053	744074	744325	yes	KC963990	cDNA skin E18	4 aa changed	252
EDQM1	-	NC_006112.2	n.a.	750893	750596	750578	750213	yes	KC963994	cDNA scale E18	1 aa deleted, 4 aa inserted	375
EDQM2	-	NC_006112.2	n.a.	754362	753920	753902	753585	yes	KC963993	cDNA skin E18	5 aa deleted	351
EDSC	-	NC_006112.2	n.a.	757613	757168	757131	756799	yes	KJ569100	cDNA skin E18	1 aa changed	333
EDWM	-	NC_006112.2	n.a.	761981	761638	761617	761139	yes	KC963981	cDNA scale adult	41 aa inserted, 2 aa changed	777
EDCH5	-	NC_006112.2	n.a.	764747	764655	764616	763999	yes	KC963991	cDNA skin E18	7 aa deleted, 2 aa changed	597
EDMPN1	-	NC_006112.2	n.a.	767684	767250	767228	767022	yes	KC963998	cDNA skin E18	1 aa changed	207
EDMPN2	-	NC_006112.2	n.a.	771404	771074	771049	770798	yes	KJ569097	cDNA skin E19	no	252
EDCRP	-	NC_006112.2	n.a.	775643	775187	775144	774512	yes	KC963999	gDNA	88 aa inserted	1158
EDCH2	-	NC_006112.2	n.a.	779952	n.d.	779606	779307	no	n.a.	n.a.	no	300
EDCH3	-	NC_006112.2	n.a.	782567	782113	782092	781793	yes	KC963988	cDNA skin E18	1 aa changed	300
EDCH4	-	NC_006112.2	n.a.	785605	785072	785051	784773	yes	KC963989	cDNA skin E18	no	279
EDQM3	-	NC_006112.2	n.a.	790619	789547	789527	789267	yes	KC963992	cDNA skin E18	1 aa changed	261
EDGH	-	NC_006112.2	795656	795633	794871	n.a.	794248	yes	KC963980	cDNA skin E18	no	648
LOR3	-	NC_006112.2	n.a.	799695	799491	799476	797560	yes	KC964001	cDNA scale E18	1 aa changed	1917
LOR2	-	NC_006112.2	n.a.	804232	803794	803779	802064	yes	KC964002	cDNA skin E18	no	1716
LOR1	-	NC_006112.2	n.a.	808797	807935	807915	806284	yes	KC963995	cDNA skin E18	no	1632
EDYM1	-	NC_006112.2	n.a.	821633	820168	820147	819704	yes	KC963982	cDNA scale adult	1 aa changed	444
Edbeta	+	NC_006112.2	n.a.	824814	825186	825202	825525	yes	KC964000	cDNA feather E18	no	324
EDMTFH	-	NC_006112.2	n.a.	830601	829926	829905	829606	yes	KC963987	cDNA feather E18	no	300
EDMTF4	+	NC_006112.2	n.a.	832239	832949	832970	833272	yes	KC963986	cDNA feather E18	no	303
EDMTF2	-	NC_006112.2	n.a.	842817	841587	841562	841299	yes	KC963984	cDNA skin E18	1 aa changed	264
EDMTF1	-	NC_006112.2	n.a.	847275	846745	846720	846457	yes	KC963983	cDNA scale adult	4 aa changed	264
EDMTF3	+	NC_006112.2	n.a.	849644	850869	850894	851124	yes	KC963985	cDNA skin E18	1 aa changed	231
Beta-keratin (cluster start)	-	NC_006112.2	n.a.	854528	854431	854410	854012	no	n.a.	n.a.	n.a.	399
Beta-keratin (cluster end)	-	NC_006112.2	n.a.	1082450	1081297	1081224	1080733	no	n.a.	n.a.	n.a.	492
EDYM2	-	NC_006112.2	n.a.	1089008	1087509	1087488	1086550	yes	KF494198	cDNA skin adult	no	939
EDQrep	-	NC_006112.2	n.a.	1094493	1093630	1093609	1092137	yes	KC963997	cDNA skin adult	2 aa changed	1473
EDPE	+	NC_006112.2	n.a.	1103327	1105053	1105074	1106024	yes	KF297351	cDNA skin E18	no	951
EDQCM	-	NC_006112.2	n.a.	1114255	1113295	1113284	1112622	yes	KF297350	cDNA skin E18	no	663
EDDM-a	-	NC_006112.2	n.a.	1121430	1120707	1120665	1118692	yes	KF716132	cDNA feather E18	2 x 15 aa inserted,15 aa deleted	2019
EDDM-b	-	NC_006112.2	n.a.	1121430	1120687	1120665	1118692	yes	KF716133	cDNA feather E18	2 x 15 aa inserted,15 aa deleted	2019
EDNC	+	NC_006112.2	n.a.	n.d.	1124136	1124144	1125064	no	n.a.	n.a.	n.a.	921

NOTE - n.a., not applicable; n.d., not determed; aa, amino acids; EDDM-a and EDDM-b are splice variants of the same gene, EDDM. Only the beta-keratin genes at the borders of the beta-keratin gene cluster are included in this table.

Suppl. Table S2B
Chicken EDC genes comprising 3 exons

	Gene predictions based on *Gallus gallus* genome sequence									Experimental data and protein predictions for *Gallus gallus* Tetra SL				
**Gene**	**Orientation of gene within EDC**	**Accession number of genomic DNA sequence**	**Start of coding sequence in exon 1**	**Exon 1 end**	**Exon 2 start**	**Start of coding sequence in exon 2**	**Exon 2 end**	**Exon 3 start**	**End of coding sequence**	**Expression confirmed by RT-PCR**	**Accession number of sequence submitted to GenBank**	**Source of sequence submitted to GenBank**	**Differences in encoded proteins (relative to prediction from reference sequence)**	**Length of coding sequence (nucl.)**
S100A9	+	NC_006112.2	739505	739513	740065	n.a.	740223	740530	740721	no	n.a.	n.a.	n.a.	360
CRNN	+	NC_006112.2	n.a.	1132656	1132855	1132880	1133017	1133140	1134156	yes	GQ149127	cDNA tongue	n.a.	1155
SCFN	+	NC_006112.2	n.a.	1138074	1139237	1139259	1139396	1139871	1151966	yes	KC700629	gDNA	n.a.	9642
S100A11	+	NC_006112.2	1153990	1153992	1154986	n.a.	1155137	1155382	1155534	no	n.a.	n.a.	n.a.	306

NOTE - n.a., not applicable ; aa, amino acid residues. Only the S100A genes flanking the region of EDKM, SEDC, and SFTPs are included in this table.

Suppl. Table S2C
Chicken EDC genes comprising 4 exons

	Gene predictions based on *Gallus gallus* genome sequence										Experimental data and protein predictions for *Gallus gallus* Tetra SL				
**Gene**	**Orientation of gene within EDC**	**Accession number of genomic DNA sequence**	**Exon 1 end**	**Exon 2 start**	**Start of coding sequence in exon 2**	**Exon 2 end**	**Exon 3 start**	**End of coding sequence**	**Exon 3 end**	**Exon 4 start**	**Expression confirmed by RT-PCR**	**Accession number of sequence submitted to GenBank**	**Source of sequence submitted to GenBank**	**Differences in encoded proteins (relative to prediction from reference sequence)**	**Length of coding sequence (nucl.)**
EDKM-a	+	NC_006112.2	747298	747837	747858	747983	748202	748306	748336	748685	yes	KJ569098	cDNA skin E19	1 aa changed	231
EDKM-b	+	NC_006112.2	747441	747837	747858	747983	748202	748306	748336	748685	yes	KJ569099	cDNA skin E19	1 aa changed	231

NOTE - EDKM-a and EDKM-b are splice variants of the same gene, EDKM.

Suppl. Table S2D
Lizard EDC genes comprising 2 exons (SEDC genes)

Gene	Gene predictions based on *Anolis carolinensis* genome sequence						Experimental data and protein predictions for *Anolis carolinensis*				
	Orientation of gene within EDC	Accession number of genomic DNA sequence	Exon 1 end	Exon 2 start	Start of coding sequence in exon 2	End of coding sequence	Expression confirmed by RT-PCR	Accession number of sequence submitted to GenBank	Source of sequence submitted to GenBank	Differences in encoded proteins (relative to prediction from reference sequence)	Length of coding sequence (nucl.)
EDPQ3	-	NW_003339570.1	84534	83145	83124	82639	yes	KF193418	cDNA dewlap	no	486
EDPQ2	+	NW_003339570.1	90356	92534	92552	93007	yes	KF193419	cDNA dewlap	no	456
EDSC	-	NW_003338916.1	1056792	1058646	1058668	1059000	yes	KC963972	cDNA skin grow.	1 aa deleted	330
EDWM	-	NW_003338916.1	1022355	1023658	1023669	1024490	yes	KC963971	cDNA skin grow.	1 aa changed	822
EDPQ1-A1	-	NW_003338916.1	1004845	1009007	1009029	1009616	yes	KC963977	cDNA skin grow.	1 aa changed, 14 aa deleted	753
EDPQ1-A2	-	NW_003338916.1	1004845	1009007	1009029	1009616	yes	KC963978	cDNA skin grow.	1 aa changed, 17 aa deleted	744
EDCM	+	NW_003338916.1	967397	965795	965770	965552	yes	KC963969	cDNA skin grow.	no	219
EDCRP	-	NW_003338916.1	944172	947046	947067	947963	yes	KC963970	gDNA	no	897
EDCQ3	-	NW_003338916.1	903173	906936	906952	907197	no	n.a.	n.a.	n.a.	246
EDCQ2	-	NW_003338916.1	845490	848566	848592	848804	yes	KF527459	cDNA dewlap	no	213
EDCQ1	-	NW_003338916.1	836666	837511	837536	837808	yes	KF527458	cDNA dewlap	no	273
LOR2	-	NW_003338916.1	821707	823476	823517	825148	yes	n.a.	n.a.	no	1632
LOR1	-	NW_003338916.1	806436	808156	808210	810338	yes	KC963979	gDNA	n.a.	2208
								KC963996	cDNA dewlap		
EDCC3	-	NW_003338916.1	764824	766864	766884	767162	no	n.a.	n.a.	n.a.	279
EDCC1	+	NW_003338916.1	761018	759802	759782	759360	no	n.a.	n.a.	n.a.	423
EDCC2	+	NW_003338916.1	750529	749409	749389	748940	no	n.a.	n.a.	n.a.	450
Beta li-ac-40	+	NW_003338916.1	737643	739466	739487	740629	no	n.a.	n.a.	n.a.	1143
EDSCP	+	NW_003338916.1	285608	283193	283171	282473	yes	n.a.	n.a.	no	699
Beta li-ac-1	+	NW_003338916.1	193285	192282	192261	191947	no	n.a.	n.a.	n.a.	315
EDPSQ	-	NW_003338916.1	n.d.	169895	169922	171817	no	n.a.	n.a.	n.a.	1896
EDEPK	-	NW_003338916.1	156653	158332	158353	158805	no	n.a.	n.a.	n.a.	453
EDPKC	+	NW_003338916.1	n.d.	134653	134615	132786	no	n.a.	n.a.	n.a.	1830
EDSPR1	-	NW_003338916.1	111589	113058	113100	113477	yes	KC963973	cDNA skin grow.	no	378
EDPCS	+	NW_003338916.1	95596	93871	93833	93222	no	n.a.	n.a.	n.a.	612
EDCPGS	-	NW_003338916.1	n.d.	78061	78079	78882	no	n.a.	n.a.	n.a.	804
EDCP	-	NW_003338916.1	63381	65846	65869	66555	yes	KF270204	cDNA dewlap	no	687
EDSQ	+	NW_003338916.1	36680	34690	34659	33964	no	n.a.	n.a.	n.a.	696
EDEPT	+	NW_003338916.1	28456	25211	25186	24629	yes	KF385879	cDNA skin 1	3 aa changed	558
EDSPR2	-	NW_003338916.1	15803	18238	18266	18451	yes	KC963974	cDNA skin	no	186
EDSPR2	-	NW_003338916.1	15803	18241	18266	18451	yes	KC963975	cDNA skin	no	186

NOTE - n.a., not applicable ; n.d., not determed; aa, amino acid residues; EDPQ1-A1 and EDPQ1-A2 are alleles of the same gene. Only the beta-keratin genes (li-ac-1 and li-ac-40) (Dalla Valle et al., 2010) at the borders of the beta-keratin gene cluster are included in this table.

Suppl. Table S2E
Lizard EDC genes comprising 3 exons

Gene	Gene predictions based on *Anolis carolinensis* genome sequence									Experimental data and protein predictions for *Anolis carolinensis*				
	Orientation of gene within EDC	Accession number of genomic DNA sequence	Start of coding sequence in exon 1	Exon 1 end	Exon 2 start	Start of coding sequence in exon 2	Exon 2 end	Exon 3 start	End of coding sequence	Expression confirmed by RT-PCR	Accession number of sequence submitted to GenBank	Source of sequence submitted to GenBank	Differences in encoded proteins (relative to prediction from reference sequence)	Length of coding sequence (nucl.)
S100A9L2	+	NW_003339570.1	n.a.	46087	47041	47059	47202	48089	48259	no	n.a.	n.a.	n.a.	315
SCFN	+	NW_003338916.1	n.a.	4640	2817	2795	2658	1926	n.d.	yes	KC700627	cDNA toe	3 aa changed, 1 nt inserted	902
S100A11L	+	NW_003339599.1	76400	76379	75736	n.a.	75578	73631	73482	no	n.a.	n.a.	n.a.	321

NOTE - n.a., not applicable; aa, amino acid residues; nt, nucleotide. Only the S100A genes flanking the region of PGLYRP3, EDKM, SEDC, and SFTPs are included in this table.

Suppl. Table S2F
Lizard EDC genes comprising 4 exons

Gene	Gene predictions based on *Anolis carolinensis* genome sequence										Experimental data and protein predictions for *Anolis carolinensis*				
	Orientation of gene within EDC	Accession number of genomic DNA sequence	Exon 1 end	Exon 2 start	Start of coding sequence in exon 2	Exon 2 end	Exon 3 start	End of coding sequence	Exon 3 end	Exon 4 start	Expression confirmed by RT-PCR	Accession number of sequence submitted to GenBank	Source of sequence submitted to GenBank	Differences in encoded proteins (relative to prediction from reference sequence)	Length of coding sequence (nucl.)
EDKM	+	NW_003339570.1	n.d.	71464	71486	71623	74056	74157	74190	n.d.	yes	KJ569101	cDNA dewlap	2 aa inserted	240

NOTE - n.d., not determed; aa, amino acid residues.

Suppl. Table S2G
Lizard EDC genes comprising 5 exons

Gene	Gene predictions based on *Anolis carolinensis* sequence												Experimental data and protein predictions for *Anolis carolinensis*				
	Orientation of gene within EDC	Accession number of genomic DNA sequence	Exon 1 end	Exon 2 start	Start of coding sequence in exon 2	Exon 2 end	Exon 3 start	Exon 3 end	Exon 4 start	Exon 4 end	Exon 5 start	End of coding sequence	Expression confirmed by RT-PCR	Accession number of sequence submitted to GenBank	Source of sequence submitted to GenBank	Differences in encoded proteins (relative to prediction from reference sequence)	Length of coding sequence (nucl.)
PGLYRP3	+	NW_003339570.1	61825	63203	63263	63311	63697	63907	65127	65245	66003	66184	yes	KC963976	cDNA skin	1 aa changed	561

NOTE - aa, amino acid residues.

Suppl. Table S3
Primers for 5´ RACE

Species	Gene	Primer	Primer sequence
Aca	*LOR1*	SP1	CATTTGCTTCGTTTGGCTAAG
		SP2	GCCACCTGAGCCACCTCCT
		SP3	AACTCTGACGTCGACGAGAAC
Aca	*EDCRP*	SP1	AAGGACCCCATACACGAAGAG
		SP2	GGTGCATTGAAAGCATAGCTC
		SP3	CACAGCAGCTTTGCTTGC

NOTE - Primer sequences are shown in 5´-3´direction; Aca, *Anolis carolinensis* .

Suppl. Table S4
PCR Primers

Species	Gene	Forward Primer	Reverse Primer
Aca	*CASP3*	CAAGGATGATCACCACAAGAG	CCTGTCTGCAGATGACAAGAG
Aca	*EDCM*	CGAACTTCCTACACTTGAGCA	TGGCAAGATGAGCACAAAAG
Aca	*EDCP*	GGTCCATTTGATCCCTTTTG	TTGCATATCACTGGCTCTGC
Aca	*EDCQ1*	CTGATACACATTGACCTTTTG	CTTTCTCTGTTGTGGCAAGAT
Aca	*EDCQ2*	ACACTTCTCAGGCAGGACTTG	GAATACCGCCAAAGCGTGCAA
Aca	*EDCRP*	TTGTCTTACTACCACTTACACGC	CTTTGCTTGCAACAGGGGGT
Aca	*EDEPT*	CCCGGCACCAAGAAAGTT	GGTACCAGACGGATCCAAGA
Aca	*EDKM*	AGAACATCAAGGAACCCAAAG	CATTTATGGTTGCTCTGCATG
Aca	*EDPQ1*	CAACAGACGTATTCCGCATC	GCTGGAGCATGAGAAGGAAG
Aca	*EDPQ2*	AGACGCAGCTCTTCCTTCCT	ACACTTTGGATCCTGGCATT
Aca	*EDPQ3*	CTTGATTCTGCCTTCCTTGTG	GGGGAGGGACAGTTTTCTTG
Aca	*EDSC*	CTTGACCAAAGCTTCTCAGTG	AGGAATTATGCAGTAGACGTTG
Aca	*EDSPR1*	CTTTGGCTTGGCGTTGAGT	AATGCAGGCAAGAGCTTGTT
Aca	*EDSPR2*	TTCACTCAGCTCCGTCGTG	CGTTCAACGAGGGAAGGATC
Aca	*EDWM*	CTGGTTCTAACCTCCCCTCA	ATGGGTCTCCCCCAGTAATC
Aca	*LOR1*	TCCTCTGGTTGCAGCTGCT	CTTCTACCACAGCATGAACTGC
Aca	*LOR2*	CTCTTGACACATCCATTCAAG	GAGCCTCGGATTTGTTGGATC
Aca	*PGLYRP-3*	GAGAAAGCCACCGCTCTTAG	TCGACCAAAGAGTGCAGAGA
Gga	*CASP3*	TGGCGATGAAGGACTCTTCT	CTGGTCCACTGTCTGCTTCA
Gga	*EDbeta*	CTTATTTCTGTTGCGCAGT	TTCTTTGGTCCTTCCAAAC
Gga	*EDCH1*	GACACTCCTCTTGTTGCTG	CGACCTCATCCTCAGTG
Gga	*EDCH4*	CATTCCTGTTGCTGAAAGG	GGAATTGGGGTTTCCAG
Gga	*EDCH5*	CTCAGCCATCCACCAGCA	CTCGATGTCGTGGCTGAG
Gga	*EDCRP*	CTCAACTGAACCCCTCAGTTAG	CAGCACACTGTCTTGCTCTTC
Gga	*EDDM*	CTTGTTCCTGGTGGTGAATCG	CAAGGCAATCTGCCTCAAGTC
Gga	*EDGH*	ATGTGTTTCCAATGGCTGAGG	GTCCCAGTGCTGGAAGC
Gga	*EDKM*	GACCAGCAGCTCCACTCCGAC	TAGGTGGAGTGGGGCACGTCC
Gga	*EDMPN1*	CCACTTCTCCTCACCGTTCAG	CGTATCCATGTCCATATCCAG
Gga	*EDMPN2*	CCTGTTCTCACTGGTCAACAG	GCAAGAAGCCAGGTGGGAAGC
Gga	*EDMTF1*	TCTCCTGCCTTAGCTCCTTG	CAAGCCATCTTCGTAGCACA
Gga	*EDMTF4*	CTCACCTTCCTCCTCCTGGT	GGGCTCTGTGCATTAGGAAG
Gga	*EDMTFH*	TCGCTTCTCTCGAGTTCCTC	TCACTGAACACAGCCAGAGG
Gga	*EDPE*	ACTTCTCCCCGTTGACTGGT	GGCAATGGCTCTGTTTCTTC
Gga	*EDQCM*	TCCCTCACTCACTTGTGCTG	GTAGGTCTGTCATCTGTATTTC
Gga	*EDQM1*	CCTTCCTCTCGCCTTCTTCT	CTGCTGAGAGCCCTCTGC
Gga	*EDQM3*	TCTGAGCAACCTTTCCAGAC	TGTGCCCTGCAGAGATGA
Gga	*EDQrep*	TCACAACGCTGTTCTCATCG	GCACAAGCGGTCACGTAGC
Gga	*EDSC*	CCCCTTGAGCTCTCACTGAA	CGTGTCTCCCAGCACTCATA
Gga	*EDWM*	CTACCTTCCCTCATCACTTG	GAGTGGGGATGAGTGAGAAAT
Gga	*EDYM1*	TCTTACTCTCCTCTCTGAGCT	GCATCTTGCTCTTCATCCATC
Gga	*EDYM2*	GTTGTTCTCACTGATGAAGTC	TCACAGGACTGCAGAGGTTG
Gga	*LOR1*	TCCATTGGACTTCCTTCAGC	CCAAAGGAGGACGATGATATG
Gga	*LOR2*	CTCAGAGTTGCGCTGTTGAA	CTGTGCCTGGTAGCTGGAG
Gga	*LOR3*	GCACACATCCAGCTGTGTT	GGACTGGGAAGATGACTCGT

NOTE - Primer sequences are shown in 5´-3´direction; CASP3 (caspase-3) is a ubiquitously expressed gene (Eckhart et al., 2008) located outside of the EDC; Gga, *Gallus gallus* ; Aca, *Anolis carolinensis* .

Suppl. Table S5

Number of EDC protein-derived peptides identified by mass spectrometry in cornification products of the chicken

	Beak								Claw								Feather								Scale							
	# 1		# 2		# 3		# 4		# 1		# 2		# 3		# 4		# 1		# 2		# 3		# 4		#1		# 2		# 3		#4	
Protein	I	S	I	S	I	S	I	S	I	S	I	S	I	S	I	S	I	S	I	S	I	S	I	S	I	S	I	S	I	S	I	S
EDbeta	0	5	0	5	0	9	0	0	0	8	0	5	0	0	0	0	0	0	0	0	0	0	0	0	0	0	0	0	0	0	0	0
EDCH3	0	0	0	2	2	0	3	0	3	0	3	0	0	0	0	0	0	0	0	0	0	0	0	0	0	0	4	0	8	0	8	0
EDCH4	18	9	20	7	17	4	31	0	21	0	19	0	24	0	16	0	0	3	0	4	0	0	5	0	28	0	28	0	36	5	34	0
EDCH5	0	0	2	0	6	0	4	0	0	0	3	0	4	0	3	0	0	0	0	0	0	0	0	0	7	0	11	0	10	0	6	0
EDCRP	0	0	0	0	0	0	0	0	0	0	0	0	0	0	0	0	0	0	0	0	7	0	2	0	0	0	0	0	0	0	0	0
EDDM	0	0	0	0	0	0	0	0	0	0	0	0	0	7	0	5	11	11	6	7	11	9	9	9	0	0	0	0	0	0	0	0
EDMTFH	0	0	0	0	0	0	0	0	0	0	0	0	0	0	0	3	0	0	0	0	0	5	0	2	0	0	0	0	0	0	0	0
EDMTF4	0	4	0	3	0	4	0	6	0	3	0	2	0	4	0	2	0	0	0	2	0	2	0	3	0	0	0	0	0	0	0	0
EDQrep	0	0	0	0	0	0	2	0	0	0	0	0	0	0	0	0	0	0	0	0	0	0	0	0	17	0	12	0	15	0	11	0
EDWM	18	15	20	15	30	4	39	13	26	0	26	13	24	0	21	0	0	0	0	0	0	0	0	0	60	23	53	13	64	11	47	19
EDYM1	19	18	39	21	45	8	52	18	35	3	40	13	34	0	40	0	0	0	0	0	0	0	0	0	0	0	14	0	0	8	26	0
LOR1	0	0	0	0	0	0	0	0	0	0	0	0	0	0	0	0	0	0	0	0	0	0	0	0	8	0	6	0	0	0	7	0
LOR2	0	0	0	0	0	0	0	0	0	0	0	0	0	0	0	0	0	0	0	0	0	0	0	0	0	0	0	0	5	0	4	0
LOR3	0	0	0	0	0	0	0	0	0	0	0	0	0	0	0	0	0	0	0	0	0	0	0	0	0	0	52	0	28	0	38	0

NOTE - Beak, claws, feathers, and scales from the leg were derived from 4 individual chicken. I, insoluble fraction; S, soluble fraction.

Suppl. Table S6
Comparison of SEDC genes characterized in this study and similar genes annotated in the GenBank (July 2014)

	This study	GenBank (July 2014)		Comparison: newly characterized genes versus GenBank annotations		
**Species**	**Gene**	**Gene**	**Product**	**Exon borders**	**Coding region**	**Notes**
Aca	***EDCQ1***	LOC103280362	uncharacterized LOC103280362	identical	different	ORF not defined in GenBank
Aca	***EDCRP***	LOC103280368	keratin-associated protein 4-7-like	different	identical	exon 1 not defined in GenBank
Aca	***EDSPR2***	LOC103280358	uncharacterized LOC103280358	identical	different	ORF not defined in GenBank
Aca	***LOR2***	LOC103280361	loricrin-like	identical	identical	
Gga	***EDbeta***	LOC101748031	claw keratin-like	identical	identical	
Gga	***EDDM***	LOC101751279	keratin-associated protein 10-4-like	different	identical	
Gga	***EDMPN1***	KRTAP10-4	keratin associated protein 10-4	identical	identical	
Gga	***EDMPN2***	LOC101747731	keratin-associated protein 5-1-like	different	identical	exon 1 not defined in GenBank
Gga	***EDNC***	LOC101751222	mucin-2-like	different	identical	exon 1 not defined in this study
Gga	***EDPE***	LOC101751113	titin-like	different	identical	
Gga	***EDQCM***	LOC101751162	keratin-associated protein 9-1-like	different	identical	exon 1 not defined in GenBank
Gga	***EDWM***	LOC425969	loricrin-like	identical	identical	part of coding sequence different
Gga	***LOR1***	LOC101747961	probable serine/threonine-protein kinase kinX-like	identical	different	
Gga	***LOR2***	LOC101747853	loricrin-like, transcript variant X1	identical	identical	
Gga	***LOR3***	LOC101747796	loricrin-like	different	identical	exon 1 not defined in GenBank

NOTE - Gga, Gallus gallus; Aca, Anolis carolinensis.

A

>Aca_EDCC1
MSCCGGGCCGGCCGGGYGGGYGGSHGGAGGGGGSFVGYTLGRDIHVTPGECYMITPGPYLRDCCGVSVCCHCVCVPDCCPCGS
GGYGGYGGYGGGCGGGGLEVDIIGESRNITMPATYLCSRVHCAESCCTCCEPCCCTC

>Aca_EDCC2
MACCGSGCCGDCCGGGYGGYGGYGGGYGGDGGGVLAAVGVNKNVHVRDHGCTMVFPTPYITNNGDFRMCCREVCAHECCKPCC
CGGGYGGYGGGDGGYGGQGPGLRVDMRQAGSTVHVPGSYLFSCYPCASSCTECCDPCCCGSCCGPC

>Aca_EDCC3
MACCMSSCTSHGGGLGSNNDDGAVLVGWTSDKVVRVSPGRSSLLIPPPSAKTTNQPFRISCHIVRCLGVGNGEMVWLNRCNYG
LIHSTLGVR

>Aca_EDCM
MSCCSRCGARSGCCCCHRSKPRVVYYVRRQVPMVPCCGGGQYSYSYVPARSCCCAPRPRLPIQQCTMPVKKY

>Aca_EDCP
MSYQCKQRCLPPPIWVKGNSIKCAEVCAPISKSPCKSVCSSDPCGSSCTTAKDLCGAPCQPVCCDPCVKVEKCPDPCCAKAEP
VICKSPCVSVCPAPCSPEPVKCKSPCASVCDATCEEAACGCQGQSGVCQPQSCFPFCVAPCQGQSCLPFCGNPCQGQSYIPVY
IPGCGVVLVPQSCCLNPCASSCPCCQPPCCCPVPSCCNPSCCSQPASCAKCSCGSCPKKQSS

>Aca_EDCPGS
MPYCQLQSNQTSAAPSGCVKSYTTECPQLCHPQCGPQCGPTCGCHGGPNCGCPCGPNCRCPCGPSCGCSCGPSCGCHGGSNCG
CPCGPSCGCPCVPCCGCPCGPSCGCQSGPNCGCPCGPSCGCRGGPNCGCPCGPNCKCPHNPTCGYPCGPSGGASCSPICNQPC
GPSCGYPCHPSCGPSWAPAGGKSCGPTCAPSGGTSCGPSCGKTCVPSGGISYGPSRKTSCSPNCGQTSGSSCAATCGSSSAIK
SEKKCDTKSKDSCSTKEK

>Aca_EDCQ1
MCSRGDRDCHKPPQQEAPSCHRGRSSCHEEKERSCHNRESSPGCGSRQPSCGAKPVPSCQQQTPGVVVPPPCQQQQQVKQPTQ
WPSQNQK

>Aca_EDCQ2
MCSRNDPAPQKKNPFPQEEEERSSCGKRETSSSCCSRKPSEEPPAPVSPPSCQEKQQVKQPTQWPPQNAK

>Aca_EDCQ3
MGRCCRCCHRERCGCGSRHGCEHECERTTVLCCKPPCRVPPPCMPPVQCGVPPMQCVQSCQLPAQVKACIPAQKSCCQTKP

>Aca_EDCRP
MSCNNCCSCCCTPCCKQSCCKQSCCQQCCCTPCCKQSCCDPCCKTKCCEPCCKTKCCDPCCKTKCCEPCCKTKCCDPCCKTKC
CDPCCKTKCCEPCCKTKCCDPCCKQKCCDPCCKQKCCDPCCKQKCCDPCCKQKCCDPCCKQKCCDPCCKQKCCDPCCKQQCCE
PCCKPQCCEPCCKPQCCEPCCKPQCCEPCCKPQCCDPCCKPQCCDPCCKPQCCDPCCKPQCCEPCCEPCCKPQCCDPCCKPQC
CEPCCEPCCKPQCCEPCCEPCCKPQCCEPCCCQTVCKPCCPPCCKTKKC

>Aca_EDEPK
MSTEETDQQKKQSSTLPPALSTFSSEPIPDREEPALPPPNEPEVPPEEEKKEEPNEKKESPPQPDTEPDPESETSKPDISSGN
EREKPPPTEQQPEEPPTLPRALSQETLKVDRSSPLEVTEDVSNQAPPSVPVEDKQQQRKQPSTWPLK

>Aca_EDEPT
MSYQARQPCTAPPIYQETSAPPYIPQCSDPSPCSEGASASSYQYTQPSVPPPCCPQSSPTEILDASTSPCVSFHTVIDGETLT
EVSSPTPCESATTQPQPTLPQPSPVEIRETPISPCVAVYATEPLEEPEAPREEPTRNYVMPGSIVILDPSGTITVKPGPDDEE
ECAGARESVFVAPYTPHFS

>Aca_EDKM
MSRLIRAFTDMMEGNHKSNPKKVKEAETFKKSEFKKLIQQELSPVQRSSSSKYKNMKNALDSDAELMTDKETVPCAY

>Aca_EDPCS
MSFFPESVEVFQPILLDGGNLVLVKQTPDCGPLKSSYPLRSLGSPSKYISHCPCPCPCPPSCLSPCRPRCLPNCGPCYGRPQC
LSPCRPICSSPCRPICPYPCQPSCVSPCYPCYQRPQFRPICVNPCQSPCSVSCPYPYSPLCPPSCIPNCYPQCQYPRSSICVQ
PACVYPVGQVSCSPICGYPCSPRGCYPYENFEVYYLG

>Aca_EDPKC
MASHNEFQSKQKHSLPPALCKKAPEPTPCPPQEDAPPPEVHQCKQTISFPPAMWPHKTPSPQEKPPCKESPVVAAPNPCSPQP
EPCKHPSIVTVPTPCPDPVPEKKSPCKEKPTEIVPIPCCPKDSPSPQEKPHCKEAPAHVGPTPCSQQKSPSKESPCCPQPEAV
PCPQQKQPCKETPVESEPTPCPQHKPPTKDSPCCPPPEPVPCAQEKKPCKETPVEVVPTPCPQQKQPLKESPCCQSPEPASCA
QEKQPCKEPPVHIVPTPCSQQKSPSKESPCCPQAEPVPCAQEKQPCKEPPVHTVPTPCPQQKQPPKESPCCPTPEPAPCAQEK
QPCKVPPVHIEPVPCPQQKQPCKETPAEVVPTPCPQEKPPTKDSPCCPPPEPVPCAQEKQPCKETPVEAVPTPCPQQKSPSKE
SPCCPQAEPVPCPQQKKPCKDTPIVTIPTPCPDPVPGPEKKSPCREPPKEVVPTPCCSKPIPSPEEKPHCKEPPAHIVPTPCA
QQKKLPKESPCCPTSEPAPCAQEKQPCKVPPVHIAPTPCPQQKPPAKESPCCSQPEEEPPCKQPPKTTVPTPCPEKRSSCKEP
TPTAPSTKCPPPIEQHQKKQPCYWPHHK

>Aca_EDPQ1-A1
MSSSGFNSCEDACNDYGSYMVSGSCGGCSGGRSLCSEPSCGASGSSCCSGRSRTMGSCCRPPSQQYCPPMQKYCPPVQQYCPP
VQKCSLPQQKYCPPVQKCSPPQQKCCLPVQKCSPPQQKCCPPVQKCCPPQQKCCPPVQTCYPPQQKCCPPVQTCYPPQQKCCP
PVQKCCPPQQYCPPVQTWYPPQQKSCPPVQSCGPPVQQSYAPSQQCCSSTQQYSQQGHQGYVTIGGPAICQTKQVCAPPGLLK
K

>Aca_EDPQ1-A2
MSSSGFNSCEDACNDYGSYMVSGSCGGCSGGRSLCSEPSCGSSCCSGRSRTMGSCCRPPSQQYCPPMQKYCPPVQQYCPPVQK
CSLPQQKYCPPVQKCSPPQQKCCLPVQKCSPPQQKCCPPVQKCCPPQQKCCPPVQTCYPPQQKCCPPVQTCYPPQQKCCPPVQ
KCCPPQQYCPPVQTWYPPQQKSCPPVQSCGPPVQQSYAPSQQCCSSTQQYSQQGHQGYVTIGGPAICQTKQVCAPPGLLKK

>Aca_EDPQ2
MSYQQQQCKQPCQPPPSQCKKTCPPQQEVCPPQQKVCPPPQQECPPPQKVCPPPQQECPPPQKVCPPPQQECPPQKVCPVPQQ
VCLPPQQECPPPKQECPPQKMCPAPQQVCPPPQQEYLPPQQECPPPQQVCPPSKQGCLPQQCQDPKCC

>Aca_EDPQ3
MSSDSFQCTQPCKAPPQCKKTVPPPQECCPPPQKCCPPPQECCPPPQKCCPPPPQKCCPPPQECCPPPPQKCCPPPPQKCCPP
PPQQCCPPPPQQCCPPPPQQCCPPPPQQCCPPPPQQCCPPPQECCPPPQKCCPPPEQCCIPQCPPPQQCCPPQNPKGF

>Aca_EDPSQ
MYCTDQQCKQACLPPPSYQKIVGTQDISGCGPRYRCQRGQSYTPQCISPCPPQQRHSYVPVSPQSCSPQRQPSYPLQHRPTFV
PKWIQPSCPLQHRPAFAPDCIQPPRPLQCRSSFSPDSIQPVPPLQRRSSFSPDTIQPVPPLQSRPSFAPDCIQPPSPIQNQPL
LAPDHLQPASPPHSKPSCSSDSIQPSYPHQGLPSIVPDHIETTCPPQRQPPCSSDTLQPSYPPQRKPSFTPSYPPQSQPPCSS
DTLQPSYPPQRKPPCSSDTLQPLYPPQRRPSFTPSYPPQSQPPCSSDIQPLYPPQRKPSFTPSYPPQSQPPCSSDTLQPSYPP
QRKPPCSSDTLQPLYPPQRKPSYTPLYQPQQKPPCSSETLQPSYPLQRRPSFTPSYPPQSQPPCSSDTLQPSYPPQRRPSYTP
PWIQPCSSQGEASYEPPCPRPSFPERPLSPPQSILQPYASPQKYQYTQKPCCPPCKSVCFGSQQTRMPQAVQTCAPKGKCVSQ
GFLGSQCSQGAIKGNQQGVIKGGQQYGSQQCIKKGGPVGITKSVQQCACQPGIMKGGLCGQQYGSQPFITKGVSAGKAMAIQQ
GASQAYVTKGGPGFAVKSSPQWSSQPCISKGGPMYATKGSQGYIRKGTKW

>Aca_EDSC
MSQQVQRGSSCCCSACCGGSRSGGCCSSSRSSGGCCSGRRSSGGCCGSSRSSGGCCGSSSGGKYVQQAQASAGCGSSSGGCCG
GSSSSGCCSGGLSQQKQVKMCPQQLK

>Aca_EDSCP
MVSGCSIPSCIAPSGSTRSVTRRISGGISSGNYSCGPVQHYAVRSCSIPYYSIQPLSCQPSCGISSCNYSCGPVEHCVVRPCP
VPYYSIQPLSCQPSCGISSCNYSCGPVEHCVVRPCPVPYYSIQPLSCQPSCGISSCNYSCGPIEHCVVRPCPVPYYSIQPQSC
QPSCGFSPCNYPCGPTESCGVQSPSIQYYPIQSVSCQPSCGVQSSCAPSCYTPIQVQSCNPCCSPC

>Aca_EDSPR1
MACPHQQCKQPCLPPPLCGKGCLPKSGKGCPPIGPCVPSCSGQSTCVSPCSPPCTKKSPCTSPCSKVLPCCSPCAPCSPTCAA
PPCAPKCCPQQHVVPCVPAQAVCPPKCAQACAPLSPCSKKSH

>Aca_EDSPR2
MSQQCKQGCKAPPCMPKCAPKSSPCCSPSAAKCPPKCNPCCPPQTVVKCPPKCKQPPKGCC

>Aca_EDSQ
MSYQVKQASLPPPTYGKKNTATATPSQAPSAPSVCTPSVCATPDSSLCMTPSQSQYGSSGCQCGSQGKSQQWQRGSMGHDSSR
TPQQPYSQCSSPMVCATPCSSLCFSQGQSQGQGQSQGQGQGQGQRPPVFRDQYGTICVLPQQLQTVTVCQDPCGNISVIPQKR
DPGKNAGTSVCGSC

>Aca_EDWM
MCESRIYAAGREPYFNLNSTWYDPAGSWLDTRRKPFHYTVNTSCVPCCNKNNNCDVPRRGGHNYRCYSYRQSTCTPECNPRLP
CGFRNPSGGPRDYWGRPIGDSCDGRTGGYYSNEESVNGSCCRASGGCGSGGGACAKPSSSIGGCGGGVCAEPGCQSSGRCGGR
RRGLCSEPGCGLFRRRRSVCSETCSRSSRGCGSGGCAGPQISFSGGCGGRGLCSEPGCGIARRRQSVCSETYSRSSRGCQPYA
RGACVGPQSSVSGGCGARGVCSEL

>Aca_LOR1
MSGQQRQSSGCCGRGGGGGGGGGGGSSCCGRRSGGSSSSSSSSCCCSSSRGSSGQQSQGSMCCGGGGGGGSGGGGSGGQKIII
VSSGGSSGGGQCCCGGGGGGGGSSSGGGSGQIIISSGGGGSGGQSSGSCGGGSGGGSGGGMQQKIPCCGGGGGGGSGGIKIIG
GGSSGGGGGGGGGGGGGSGGMKSMGGGSSGGSSCGMGGGSGGGGGSGGGGGSGGVKIIGGGSSGGGGGGGGGGSGGMKSMGGG
SSGGSSCGMGGGSGGGGGSGGGGGSGGIKIIGGGSSGGGGGGGGGGGGSGGMKSMGGGSSGGSSCGMGGGSGGGGGSGGVKII
GGGSSSGGGGGGGGGGSGGMKSMGGGSSGGGSSGGSGGKTIIICGGSSGGGQSSGGQSSGGQSSGGQSSGGQSSGCCMGGGSS
GSGGGGGQTIIVPCGGSSGSGGQSSGGQSSSSCMGGGSSGGGGGGGGGGGGGGQNSGHQTIIVSCGGSSGGGGQSSRRRQSSG
GQSSSSCMGGGSSGGGGGQSSGCCMGGGSGGGGGGGGQTIIVPCGGSSGSGGQSSGGQSSSSCMGGGSSGGGGGGGGQNSGHQ
TIIVSCGGSSGGGGQSSGGQSSGGQSSGCCMGGGSSGGGGGSGGQTIIVPCGGSSGGGGGGQSSGGQSSSCCMGGGSSSGGGG
GGSGGQTIIIPCGGSSGGGGGGSSGGQSSGCCSGGGGGSSGGSSMQSKQSFSIPACLSQTKQMNTWPSGQK

>Aca_LOR2
MSQQRQSGCCGSNRSPRCGRRGSGSGSSCCGGSSGGGSSCGRRGSGSSGCGGSSVGIAQVSGGSSCGRRGSGSSCCGGSSVGI
AQVSGGYGGSCCSSGGGSGQGVVIVSGGQGSSCCGSSGGIQQIRGSGCCSGGSSGGAVIIPAGVGGQSSGCCVGGGYGSGMGQ
QKIPVIDSGFGSGGIGCGGGSGGGLGGQSIIVPVPGSGGSGGCYGGGQQVIGSGISSASGCCSGDAGVGGVKVIGGGSGRVSP
VCGGGVSGGGVKVIGGSGRVSPVCGGGVSGGGVKVIGGGSGRVSPVYGGGASCGGVKVIGGGSGRVSPVYGGGASCGGVKVIG
GGSGRVSPVYGGGASCGGVKVIGGGSGRVSPVYGGGVSGGGVKVIGGGSGGGGSICGGGVSGGGVKVIGGGSGGGGSICGGGL
SDGGVKVVGGGSGRISPVYGGGVSGGGVKVVGGGSGGGGSICGGGLSGGGVKVIGGGSGRVSPVCGGGSGGQTIVVSGGSSGG
CCGGASSGTSVVVGGGGSSQVKVPLVVPPCLGQTKQVTSLPPSCK

B

>Gga_EDbeta
MTHSLSPCTDGSPSLCGVAVPQPVANSCNEPCVVQCPDSTVVIYPPPVVVTIPGPILSTFPQQSTVGSVGAPEVASGLSSAPA
PGGLQVSGGARWARGYPVGSCQPC

>Gga_EDCH1
MGSSESCHSQNPSCHSSHACNDSGPSCHSSSQNIPAPXYACVXPCSPPAQTYCVPTQCYPPAVQTCPQNTNAACNLPPPCPKF

>Gga_EDCH2
MCSRGSSCHSHETSCHGSRSSCHDSGPSCHYTIQRVSVPKYTYMTPCCPPVQTFCPPVQRYCPPVQPCCPPVKTYCPPIPYCP
QYTKNICKLPPPCPKF

>Gga_EDCH3
MCSRGSSCHSHETSCHGSRSSCHDSEPSCHYTIQRVPVPKYTYMTPCCPPVQTFCPPVQRYCPPVQPCCPPVKTYCPPIPYCP
QYTKNICKLPPQCPKY

>Gga_EDCH4
MCSRGSSCHSHETSCHGSRSSCHDSEPSCHYTIQRVPVPKYTYVTPCCPPVQTFCPPVQRYCPPAQTYCPPVPYCPQYTKNIC
KLPSSFPKY

>Gga_EDCH5
MQYRKGEDNVDVCHDDTTCGCHDGPVSIPDLSPCHDRSSLSHDIEGSCQSVPQGCQPIEPCPPRGCCPAQRSWDCGKPRRRVE
VSPVQPVCPPVKIHRRPLQQYRPPLQSEEQGCCKPRRRVEQCPSEEPITLHPLPLQHRCPCIPYCRPPVQHCRPPVQHCRPPV
QHCCPTAVPLPQHPGQHQCKQVHILPPFQQKK

>Gga_EDCRP
MCPNVCCSSGCCSVVKSKTVCCSPCQKTVCCDPCQKTVCCEKTVCCDPCQKPCCDPCQTCCDPCQKPCCDPCQTCCDPCQKPC
CDPCQTCCDPCQTCCDPCQKPCCDPCQQSVCCDPCQKPCCDPCQTCCDPCQKPCCDPCQTCCDPCQKTVCCDPCQTCCDPCQK
TVCCDPCDPCQTCCDPCQKPCCDPCQTCCDPCQKPCCDPCQTCCDPCQKTVCCDPCQKPCCDPCQTCCDPCQKTVCCDPCQKP
CCDPCQTCCDPCQTCCDPCQKTVCCDPCQKTVCCDPCQKPCCDPCQTCCDPCQKPCCDPCQTCCDPCQKPCCSCCSQQCLQTC
CRPCCCSGRLSCCSYVVKKKPVVVCCSPVQYCSPLRKCCVPIRQCCTTVKKCC

>Gga_EDDM
MLCQTDCQRGLPCLPPHVVLVRNLPVSRSVDPCNSVCSVSRLNTCADPCYYARVPQGTTTYLKLGSCDLRQIALDPCCLGITT
LTDPCCQDVTRCSSTRCVDPCCCKVTECSSTRYVDSCCQDATQCTTRCVDSCCQDATQCTTRCVDPCCQDVTQCTTRCVDPCR
QDATQCTTRCVDPCRQDATQCTTRCVDPCCQDVTKCTTRCVDPCRQDTTQCTTRCVDPCCQDVTKCTTRCIDPCRQDTTQCTT
RCVDPCCQDVTRCTTRCVDPCCKEVTRCTTRCVDPCCQDVTKCTTRCVDPCCKEVTKCTTTRCVDPCCKEVTRCTTRCADPCC
GEVTKCTTKYVDPCCRPVTRCATTCVDPCCGRVTKCTNKCVDPCYGAVTRCSTKCVEPCCEEVSKCTSRCVDPCCREVTKCTT
RCVDPCCGRVTKCARYKNPCCGEVSKCTTKCVDPCCGRVTKYVDPCCREVTSCKTRCVDPCCKEVTRCTTTCVDPCCAEVTRC
ATKCIDPCCQDATKCTTTCVDPCCREVTKCTTTCVDPCCQEVTKCTTTCVDPCCKEVTKCTTTCVDPCCQEVTKCTTTCVDPC
CREVAKCTTTCVDPCCQDATQCTTRCADPCCQDVTQCTSTCVDPCVPPCFVRPTPLCASICGRHYSISCADICCRK

>Gga_EDGH
MCFQWLRKIFQFLRGRCSRQNRGYSNDISSHYGGTPRLQGRGSFFCSGEGSIIYSRGAAPTYSPYSVTAGCTPGSDGVVVISG
GDNEGASGWSTMGGTVPTYSPRGAAGYGSEDAGWGMVGGSAGGGGSGCYSGKLGVGRGSGYGYDAPQRQKAVTTSAGQGLYGG
ASGYGTGGELSSGGAGMAQVMQQKCPVVIPDIEPQQIKQSSQWPPSQKK

>Gga_EDKM
MSRLIKAITDMMDGSARRGKKSESFSRSEFKKLIQQEFAPVKRSSTSKYHYIGSPLDSDTEPMNKKERGSSKTCVY

>Gga_EDMPN1
MPNGGCGCCGGNGNNXYTVCCYSSPRCCKTPCCYPSQYCCPTPCCMPMTCCYTLSSNSNNSGCCGCGN

>Gga_EDMPN2
MPNGCCGGGSNCTVCCYSSKSCKTPCCKTQSCCPSQCCYPSQCCCPSQCCCPSQCCAMPCCMPMTCCYTTSNSNNSGCCGCGN

>Gga_EDMTF1
MTFCYQNQWEDSCYSPCSYRTCDWGSWGSPCGYRSYGWGSPCGYRGSWWLSGCRDWCPSYSSRWYSPWSTRCTRRYSVSSCSP
CSSW

>Gga_EDMTF2
MTFCYQNQWEDSCYSPCSYRTCDWGSWGSPWGYRSYGWGSPCGYRGSWWLSGCRDWCPSYSSRWYSPWSTRCTRRYSVGSCSP
CSSW

>Gga_EDMTF3
MTFCYQNQWEDSCYSPCSYRTCDWRSWGSPCGFRGSWWLSGCRDWCPSSSSRWYSPWSTCYTRRYSVSSCSPCSSW

>Gga_EDMTF4
MTFLHDDCYFPHSYRGLHYSSPFNYRGFGGLYDFWDRYGHDGLYGHWGFCGSRDHYGFGGLNSGHRWLYGDWYGYPSWYGSRH
GHHFGSRYGQRYGYWGW

>Gga_EDMTFH
MTFHREFYNDEHYSPFCQEDLHGLWGLNDHRFKHLYGLHRDHHHDYNQHWSPYGYNRSFGSLYGNRSLSSHGGYYGHGDFFGF
GHRHPYFSQFGHRYWY

>Gga_EDNC
MSEPCELPCLPPPACVGTSVPTDLCTSCSVTVHSDTCPTQDPCPCPEQTPGVDLCQAAPSCPDVCGPACPPPTVPAPGDICDP
CTSQCVTSCVCPAADLQQCQAETSTSLQSTSPAAPCAPETVGMCMEAFPHQHAASPCTPETDEIYVETILVQHPPLSTKLCTP
EAAGPCVETCPSQHGIAPAELCILETTTGTCMETPSPQHSESLCTIEVLEACTETPPTICMEDPSCPQAASTPHPEQCPLPCT
DPCTVQTSVCTEPCSSAATQGPSSASSPRAPFRLNTRTAAIVERCLARCQSWLRGTK

>Gga_EDPE
MQCKQEVTLPPGLGKTISKQNQEPEPCPEPVPCPQQHQPEAQVAEVIAAVVVQYPEETEPLPAPSVEPGKGEAPAVVIPECPP
QEQQQQQSKLPPTLPPVSQPEPVACSQEKSACKELPEPVLAVCPEAVPQQKQECKEIPVQVPVISPEPAKCPQEKQEHKEIPV
PVPVQNPEPVPCPQEKQECKEVPVPEPVQEPEPAPCPQEKQECKEIPVVVPVQNPEPAPCPQEKQECKEVPVPTPCPAEKQEC
KEIPVVVPVQNPDPVPCPQEKQECKEVPVPDPEPEQSSIPEKHPPLEQQQVKQPSPWPLTQK

>Gga_EDQCM
MSYYEQCKQPCLPPPICLQKCSQSVEPCSNICVKPCPTQCVEVCQNPCASECTTCCSTQCVEPCSTQCSTQCVTQCVEPCSTQ
CVTQCVEPCSTQCSTQCVEPCSTQCSTQCVTQCVEPCSTQCGTQCVTQCVEPCSTQCTTQCVDVCPPTCVDACIKPCATQCPT
QCPVPCVEQCSTQCSTKCVEPCPAPCVEVCTKKCVDTCETVCLEPCGTVCTRPC

>Gga_EDQM1
MCSRQDRDQCHSQERYTRQSSGCHSSGGGGCHSSGGGGGCHSSGGGSGGCHSSGGGGGCHSSGSSGGCHSSSGGGGCHSSGGS
SCHGKPQVQYHYYHHQQQQQQQQQQQQQQQQVHQLPSQKMK

>Gga_EDQM2
MCSRQDRDQCHSQERYTRQSSGCHSSGGGGCHSSGGGGGCHSSGGGSGGCHSSGGGGGGCHSSGSSGGCHSSSGGGGCHSSGG
SSCHGKPQVQYHYYHQQQQQQQQVHQLPSQKMK

>Gga_EDQM3
MCSRADRGCHSSESSSCHSGGSSCHGSEEVTCHEVSAVQDGTPVVVLQPQCPVVTVPTQGPVAPVPCQQQQQQIKQPVQWPTQ
QQK

>Gga_EDQrep
MLCYQQQCFPPLHQEHIPIKCPQTHPLMHSWQSELCTPQYVTPCVPRQRFLSSSFSPQQCVTQCIPRQQYATKCVQQQQCVTQ
HIPPARCVTTCVPQQSCAAQGMSQEPCVTKCMPQQQCATKCISQQQCATKCIPQQQCAARCVTTCIPQQPFLTKGIRQQHSAT
VCIPQHCVTTYAPHEQCATRCVTTCVPQQRATRCVSQRYVTACAPQQCANKSIPQQQQCATKCIPQQQCATRCVTTCVPQPCE
TKGTSICVPQQQCATKCIPQQQCVTKCVPQQCATKCIPQQQCATKCIPQQQCATKCIPQQQCATKCIPQQQCAKCIPQQQCVT
KCIPQQQCVTKCIPQQQCVTKCAPQQQCTKCIPQQQCATKCVPQQCATKCIPQQQCVTKCIPQQQCATKCAPQQCATKCIPQQ
QQCATKCVPQQCATKGIPQQHQCATKGILQQQQCVTKCVPQQSVTQCVPQQPYQSGGVKISSHAKKYCSASKWPW

>Gga_EDSC
MCSRQDKDQCHRQERSSCHSSEGCGGGRGGSGCHGSSGSMCHSSSGGFGCHRSSGSGCHGSSGSGCHGSSGSGCHGSSGGSGC
HGSSGGSCHGKPQDCQQQIYQVSSKMK

>Gga_EDWM
MIYSSGRESYFNLNSTWYDPAGSWLDTRRTPFRYGYNNCCSSRCDGEGVEGMRGHNYRHYGYRQPVCSERCQGYSTAESCHGG
GGSSCARRPTYSYGSTGGCQGYGRSVCSERCQGSSGSCHGGGGSSCVRRPTYSYGSTGGCQGYGRSVCSERCQGSSGSCHGGG
GSSCVRRPTYSYGSTGGCQGYGRSVCSERCLGSSGGGCHSSGQQPQCSEPVQYIPQCCPMPVPVQQVPTAKCIPHQQQQQQQV
CKVPARKIK

>Gga_EDYM1
MSYWYQYKQQCFIPSGAKHITPAFTQCHSPGVVKSVPCASCASRDAKQCTTRRIVQSIPKCPMPCTSSCVEKHVVEGHSSSCS
HRSADGCTMMFSQPHGQHLCMPHCRQVGVTERPQVCAPVHVGQHSSYPCSYQWSDSYHYRCGQS

>Gga_EDYM2
MHYYGERYKQLYLPGSSYVTESHQHCTSQPDACSTTCHPLSIIKSPMPRWQSYVQPFTYVCSQPSVTHSTLLPCQPCVQQCVV
LCPDPCETTCLLPCRKPSVRLSPAQPLQSCENGHLEKKRVAKSLPPCAPRCPEPGKLKFPPCGIRYSSSCKDECRSKKVSRCS
SQRYGAENRCLQGMTSGYSLPLRGVTECPPQQCVTQCFMEEYISPFPHQKCSKGYPTQECITTCTQQQVPKCPPGSGVKGSSS
QQHLAKCSLLQEGAKHKSSSAQHISKSKCLHPCAVQRSPRHHAEDVKRSSHSKKSRCASKWLW

>Gga_LOR1
MGSHQQKGEGQGISEQSGGCHGGGSGGSSCHGGGGGGSCHSSGGGGSIGYQSQGSSCHGGGSSGGGGAIYQTHISSSSFGGGG
GGGGGSSGHQGQEPICIIGGGGGSSGGGGGSSHQSQGPICIGGGGGGGGGSDHQSQGPICIGGGGGGGGGSGHQSKIPICISG
GGGGKGEGGSSHQGQGPICIGGGGGGGGGGGSSHQGQGPICIGGGGGGGGGGGSSHQGQGPICIGGGGGGKGEGGSSHQGQGP
ICIGGGGGGGGGGGSGHQSQGPICIGGGGGGGGGGGSSGHQGQGPICIGGGGGGGGGSGHQSQGPICIGGGSIGGGGGGGGSS
HQGQGPICISGGGGGGGGGGGSSHQSQGPICIGGGGGGGGGGGSGHQSQGPICIGGGGGGGGGGSGYQGQGPICIGGGGGGGG
GSGHQSQGPICIGGGGGGGGGGSGYQGQGPICIGGGGGGGSSHQSQGPICIGGGGGGGGGGSGYQSQGPICIGGGGGGGGGGG
SGHQGQGSICIIGGGSGGGGSSGSGGMSMQQQTQPISWPPQTKHK

>Gga_LOR2
MCSRQSSGGCHESSSQSGGCCSGGSSSSYQAQGSSCCGGSSGYSMGGGYSGGSGGSSQKIIISSGGGGGGSSGCCGGGSSSGG
SSGGKIIIGGGGSSGGSSGCCSGGSSSYGMGGGYSSGGYSGSKSIIGGGSSGGSSGCCGGGSSSGGSSGGKIIITGGGSSGGS
SGCCSGGSSGYGIGGGYSSGGYSGSKSIIGGGGSSGGSSGCCGGGSSSGGSSGGKIIITGGGSSGGSSGCCSGGSSGYGIGGG
YSSGGYSGSKSIIGGGGSSGGSSGCCGGGSSSGGSSGGKIIITGGGSSGGSSGCCSGGSSGYGIGGGYSSGGYSGSKSIIGGG
GSSGGSSGCCSGGSSGYGIGGGYSSGGYSGSKSIIGGGSSGGSSGCCGGGSSSGGSSEGKIIITGGGSSGGSSGCCSGGSSYG
MGGGSSGGYSGSKSIIGGGSSGGSSGCCGGGSSGYGSSGYGSSSYGSGGSGQKIIISSGGGGGGSSGCCGGGSSSGGSSGGKI
IIGGGGSSGGSSGCCGGGSSGGSGGSSGHTIIISSGGGSGGYGQSSQQKCPIVIPHIESHQTKQACYFPGQQK

>Gga_LOR3
MCSRQSSGGCHESSSQSGGCCSGGSSSSYQAQGSSCCGGSSGYSMGGGYSGGSGGSSQKIIISSGGGGGGSSGCCGGGSSSGG
SSGGKIIIGGGESSGGSSGCSSGGSSGYGIGGGYSSGGYSGSKSIIGGGGSSGGSSGCCGGGSSSGGSSGGKIIITGGGSSGG
SSGCCSGGSSYGGGYSSGSSGSKSIIGGGSSGGSSGCCSGGSSGYGTSGYGSSRYGSGGSGQKIIISSGGGGGGGSSGCCGGG
SSSGGSSGGKIIITGGGSSGGSSGCCSGGSSAYGIGGGYSSGGYSGSKSIIGGGGSSGGSSGCCGGGSSSGGSSGGKIIITGG
GSSGGSSGCCSGGSSSYGIGGGYSSGGYSGSKSIIGGGGSSGGSSGFCGGGSSSGGYSEGKIIITGGGSSGGSSGCCSGGSSG
YGMGGGYSSGGYSGSKSIIGGGSSGGSSGCCGGGSSSGGSSEGKIIITGGGSSGGSSGCCSGGSSYGMGGGSSGGYSGSKSII
GGGSSGGSSGCCGGGSSGYGSSGYGSSGYGSGGSGQKIIISSGGGGGGSSGCCGGGSSSGGSSGGKIIVGGGGSSGGSSGCCG
GGSSGGSGGSSGHTIIISSGGGSGGYGQSSQQKCPIVIPHIESHQTKQACYFPGQQK

C

>Hsa_CRCT1
MSSQQSAVSAKGFSKGSSQGPAPCPAPAPTPAPASSSSCCGSGRGCCGDSGCCGSSSTSCCCFPRRRRRQRSSGCCCCGGGSQ
RSQRSNNRSSGCCSGC

>Hsa_IVL
MSQQHTLPVTLSPALSQELLKTVPPPVNTHQEQMKQPTPLPPPCQKVPVELPVEVPSKQEEKHMTAVKGLPEQECEQQQKEPQ
EQELQQQHWEQHEEYQKAENPEQQLKQEKTQRDQQLNKQLEEEKKLLDQQLDQELVKRDEQLGMKKEQLLELPEQQEGHLKHL
EQQEGQLKHPEQQEGQLELPEQQEGQLELPEQQEGQLELPEQQEGQLELPEQQEGQLELPEQQEGQLELPQQQEGQLELSEQQ
EGQLELSEQQEGQLKHLEHQEGQLEVPEEQMGQLKYLEQQEGQLKHLDQQEKQPELPEQQMGQLKHLEQQEGQPKHLEQQEGQ
LEQLEEQEGQLKHLEQQEGQLEHLEHQEGQLGLPEQQVLQLKQLEKQQGQPKHLEEEEGQLKHLVQQEGQLKHLVQQEGQLEQ
QERQVEHLEQQVGQLKHLEEQEGQLKHLEQQQGQLEVPEQQVGQPKNLEQEEKQLELPEQQEGQVKHLEKQEAQLELPEQQVG
QPKHLEQQEKHLEHPEQQDGQLKHLEQQEGQLKDLEQQKGQLEQPVFAPAPGQVQDIQPALPTKGEVLLPVEHQQQKQEVQWP
PKHK

>Hsa_KPRP
MCDQQQIQCRLPLQQCCVKGPSFCSSQSPFAQSQVVVQAPCEMQIVDCPASCPVQVCQVSDQAPCQSQTTQVKCQSKTKQVKG
QAQCQSKTTQVKGQAASQSQTSSVQSQAPCQSEVSYVQCEASQPVQTCFVECAPVCYTETCYVECPVQNYVPCPAPQPVQMYR
GRPAVCQPQGRFSTQCQYQGSYSSCGPQFQSRATCNNYTPQFQLRPSYSSCFPQYRSRTSFSPCVPQCQTQGSYGSFTEQHRS
RSTSRCLPPPRRLQLFPRSCSPPRRFEPCSSSYLPLRPSEGFPNYCTPPRRSEPIYNSRCPRRPISSCSQRRGPKCRIEISSP
CCPRQVPPQRCPVEIPPIRRRSQSCGPQPSWGASCPELRPHVEPRPLPSFCPPRRLDQCPESPLQRCPPPAPRPRLRPEPCIS
LEPRPRPLPRQLSEPCLYPEPLPALRPTPRPVPLPRPGQCEIPEPRPCLQPCEHPEPCPRPEPIPLPAPCPSPEPCRETWRSP
SPCWGPNPVPYPGDLGCHESSPHRLDTEAPYCGPSSYNQGQESGAGCGPGDVFPERRGQDGHGDQGNAFAGVKGEAKSAYF

>Hsa_LCE1A
MSCQQSQQQCQPPPKCTPKCPPKCPTPKCPPKCPPKCPPVSSCCSVSSGGCCGSSSGGGCSSGGGGCCLSHHRRHRSHRHRLQ
SSGCCSQPSGGSSCCGGDSGQHSGGCC

>Hsa_LCE1B
MSCQQNQQQCQPPPKCIPKCPPKCLTPRCPPKCPPKCPPVSSCCSVSSGGCCGSSSGGSCGSSSGGCCSSGGGGCCLSHHRRR
RSHCHRPQSSGCCSQPSGGSSCCGGGSGQHSGGCC

>Hsa_LCE1C
MSCQQSQQQCQPPPKCTPKCPPKCPTPKCPPKCPPKCPPVSSCCSVSSGGCCGSSSGGSCGSSSGGCCSSGGGGCCLSHHRRR
RSHCHRPQSSGCCSQPSGGSSCCGGGSGQHSGGCC

>Hsa_LCE1D
MSCQQSQQQCQPPPKCTPKCTPKCPAPKCPPKCPPVSSCCSVSSGGCCGSSSGGGCGSNSGGCCSSGGGGCCLSHHRRHRSHR
RRPQSSDCCSQPSGGSSCCGGGSSQHSGGCC

>Hsa_LCE1E
MSCQQSQQQCQPPPKCTPKCPPKCPTPKCPPKCPPKCPPVSSCCSVSSGGCCGSSSGGSCGSSSGGCCSSGGGGCCLSHHRHH
RSHRHRPQSSDCCSQPSGGSSCCGGGSGQHSGGCC

>Hsa_LCE1F
MSCQQSQQQCQPPPKCTPKCPPKCPTPKCPPKCPPKCPPVSSCCSVSSGGCCGSSSGGCCSSGGGGCCSSGGGGCCLSHHRRR
RSHRHRPQSSDCCSQPSAGSSCCGGGSGQHSGGCC

>Hsa_LCE2A
MSCQQNQQQCQPPPKCPPKCPPKCPPKCRPQCPAPCPPPVSSCCGPSSGGCCGSSSGGCCSSGGGGCCLSHHRPRLFHRHRHQ
SPDCCECEPSGGSGCCHSSGDCC

>Hsa_LCE2B
MSCQQNQQQCQPPPKCPPKCTPKCPPKCPPKCLPQCPAPCSPAVSSCCGPISGGCCGPSSGGCCNSGAGGCCLSHHRPRLFHR
RRHQSPDCCESEPSGGSGCCHSSGGCC

>Hsa_LCE2C
MSCQQNQQQCQPPPKCPPKCTPKCPPKCPPKCPPQCPAPCFPAVSSCCGPSSGSCCGPSSGGCCSSGAGGCSLSHHRPRLFHR
RRHQSPDCCESEPSGGSGCCHSSGGCC

>Hsa_LCE2D
MSCQQNQQQCQPPPKCPPKCTPKCPPKCPPKCPPQCPAPCSPAVSSCCGPSSGSCCGPSSGGCCSSGGGGCCLSHHRPRLFHR
RRHQSPDCCESEPSGASGCCHSSGGCC

>Hsa_LCE3A
MSCQQNQQQCQPPPKCPAKSPAQCLPPASSSCAPSSGGCGPSSERSCCLSHHRCRRSHRCRCQSSNSCDRGSGQQGGSSSCGH
SSAGCC

>Hsa_LCE3B
MSCQQNQQQCQPLPKCPSPKCPPKSSAQCLPPASSCCAPRPGCCGGPSSEGGCCLSHHRCCRSHRCRRQSSNSCDRGSGQQDG
ASDCGYGSGGCC

>Hsa_LCE3C
MSCQQNQQQCQPPPSCPSPKCPPKSPAQCLPPPSSDCALSSGGCGPSSESGCCLSHHRHFRSHQCRRQRSNSCDRGSGQQGGG
SCRGHGSGGCC

>Hsa_LCE3D
MSCQQNQQQCQPPPKCPSPKCPPKSPVQCLPPASSGCAPSSGGCGPSSEGGCFLNHHRRHHRCRRQRPNSCDRGSGQQGGGSG
CGHGSGGCC

>Hsa_LCE3E
MSCQQNQKQCQPPPKCPSPKCPPKNPVQCLPPASSGCAPSSGGCGPSSEGGCFLNHHRRHHRCRRQRSNSCDRGSGQQGGGSG
CCHGSGGCC

>Hsa_LCE4A
MSCQQNQQQCQPPPKCPIPKYPPKCPSKCASSCPPPISSCCGSSSGGCGCCSSEGGGCCLSHHRHHRSHCHRPKSSNCYGSGS
GQQSGGSGCCSGGGCC

>Hsa_LCE5A
MSCQQSQQQCQPPPKCTPKCPPKCTPKCPPKCPPKCPPQCSAPCPPPVSSCCGSSSGGCCSSEGGGCCLSHHRPRQSLRRRPQ
SSSCCGSGSGQQSGGSSCCHSSGGSGCCHSSGGCC

>Hsa_LCE6A
MSQQKQQSWKPPNVPKCSPPQRSNPCLAPYSTPCGAPHSEGCHSSSQRPEVQKPRRARQKLRCLSRGTTYHCKEEECEGD

>Hsa_LELP1
MSSDDKSKSNDPKTEPKNCDPKCEQKCESKCQPSCLKKLLQRCFEKCPWEKCPAPPKCLPCPSQSPSSCPPQPCTKPCPPKCP
SSCPHACPPPCPPPE

>Hsa_LEP7
MCDQQKQPQFPPSCVKGSGLGAGQGSNGASVKCPVPCQTQTVCVTGPAPCPTQTYVKYQVPCQTQTYVKCPAPCQRTYVKYPT
PCQTYVKCPAPCQTTYVKCPTPCQTYVKCPAPCQMTYIKSPAPCQTQTCYVQGASPCQSYYVQAPASGSTSQYCVTDPCSAPC
STSYCCLAPRTFGVSPLRRWIQRPQNCNTGSSGCCENSGSSGCCGSGGCGCSCGCGSSGCCCLGIIPMRSRGPACCDHEDDCC
C

>Hsa_LOR
MSYQKKQPTPQPPVDCVKTSGGGGGGGGSGGGGCGFFGGGGSGGGSSGSGCGYSGGGGYSGGGCGGGSSGGGGGGGIGGCGGG
SGGSVKYSGGGGSSGGGSGCFSSGGGGSGCFSSGGGGSSGGGSGCFSSGGGGSSGGGSGCFSSGGGGFSGQAVQCQSYGGVSS
GGSSGGGSGCFSSGGGGGSVCGYSGGGSGCGGGSSGGSGSGYVSSQQVTQTSCAPQPSYGGGSSGGGGSGGSGCFSSGGGGGS
SGCGGGSSGIGSGCIISGGGSVCGGGSSGGGGGGSSVGGSGSGKGVPICHQTQQKQAPTWPSK

>Hsa_PRR9
MSFSEQQCKQPCVPPPCLPKTQEQCQAKAEEVCLPTCQHPCQDKCLVQAQEVCLSQCQESSQEKCPQQGQEPYLPPCQDQCPP
QCAEPCQELFQTKCVEVCPQKVQEKCSSPGKGK

>Hsa_SMCP
MCDQTKHSKCCPAKGNQCCPPQQNQCCQSKGNQCCPPKQNQCCQPKGSQCCPPKHNHCCQPKPPCCIQARCCGLETKPEVSPL
NMESEPNSPQTQDKGCQTQQQPHSPQNESRPSK

>Hsa_SPRR1A
MNSQQQKQPCTPPPQPQQQQVKQPCQPPPQEPCIPKTKEPCHPKVPEPCHPKVPEPCQPKVPEPCQPKVPEPCPSTVTPAPAQ
QKTKQK

>Hsa_SPRR1B
MSSQQQKQPCTPPPQLQQQQVKQPCQPPPQEPCIPKTKEPCHPKVPEPCHPKVPEPCQPKVPEPCHPKVPEPCPSIVTPAPAQ
QKTKQK

>Hsa_SPRR2A
MSYQQQQCKQPCQPPPVCPTPKCPEPCPPPKCPEPCPPPKCPQPCPPQQCQQKYPPVTPSPPCQSKYPPKSK

>Hsa_SPRR2B
MSYQQQQCKQPCQPPPVCPTPKCPEPCPPPKCPEPCPPPKCPQPCPPQQCQQKYPPVTPSPPCQPKYPPKSK

>Hsa_SPRR2D
MSYQQQQCKQPCQPPPVCPTPKCPEPCPPPKCPEPCPSPKCPQPCPPQQCQQKYPPVTPSPPCQPKCPPKSK

>Hsa_SPRR2E
MSYQQQQCKQPCQPPPVCPTPKCPEPCPPPKCPEPCPPPKCPQPCPPQQCQQKCPPVTPSPPCQPKCPPKSK

>Hsa_SPRR2F
MSYQQQQCKQPCQPPPVCPAPKCPEPCPPPKCPEPCPPSKCPQSCPPQQCQQKCPPVTPSPPCQPKCPPKSK

>Hsa_SPRR2G
MSYQQQQCKQPCQPPPVCPTPKCPEPCPPPKCPEPYLPPPCPPEHCPPPPCQDKCPPVQPYPPCQQKYPPKSK

>Hsa_SPRR3
MSSYQQKQTFTPPPQLQQQQVKQPSQPPPQEIFVPTTKEPCHSKVPQPGNTKIPEPGCTKVPEPGCTKVPEPGCTKVPEPGCT
KVPEPGCTKVPEPGCTKVPEPGYTKVPEPGSIKVPDQGFIKFPEPGAIKVPEQGYTKVPVPGYTKLPEPCPSTVTPGPAQQKT
KQK

>Hsa_SPRR4
MSSQQQQRQQQQCPPQRAQQQQVKQPCQPPPVKCQETCAPKTKDPCAPQVKKQCPPKGTIIPAQQKCPSAQQASKSKQK

Suppl. Fig. S1. Amino acid sequences of SEDC and EDKM proteins of green anole lizard (**A**), chicken (**B**), and human (**C**). Amino acid sequences were deduced from cDNA and genomic sequences determined in this study and from the genome sequences available in the GenBank. Details about coding sequences are provided in Suppl. Table S1. Amino acid residues are color-coded in line with Fig. 3. Aca, *Anolis carolinensis*; Gga, *Gallus gallus*; Hsa, *Homo sapiens*.

```
Beta core                                                  ************####################
Gga_BKfeather  ----------------------------MSCFDLCRPCGPTPLANSCNEPCVRQCQDSRVVIEPSPVVVTLPGPILSSFPQNTAVGSSAS
Gga_BKclaw     -----------------------MSCSSLCAPACG-VATPTPLANSCNEPCVRQCPDSTVVIQPPATVVTFPGPILSSFPQNAAVGSAGV
Gga_BKscale    -----------------------MSCYDLCPTNIGGISRPQPIADSGNEPCVRQCPDSTTVIQPPPVVVTFPGPILSSFPQDSVVGSSGA
Gga_EDbeta     -------------------MTHSLSPCTDGSPSLCGVAVPQPVANSCNEPCVVQCPDSTVVIYPPPVVVTIPGPILSTFPQQSTVGSVGA
Aca_BK2        -----------------------------------MTFGWGNCGYGCNVACESQCPPSHVYIQPPCYAVTIPGPVMHCDTEPCAASSHTP
Aca_BK28       --------------------------------MALCDNVCNSSLCGFNPVCVAQTPASEVLIRPAPIAVTIPGPILSASPEPAEVVQYNP
Aca_BK7        --MSCYTPSCYIPSCNTSCYPFRAYGSYGYSSLGGGLAIGGGSSNVISEGCNAKLPDSEICVQPQCMNITLPGPIVS-YSSTTKVGGCSP
Aca_EDSCP-N    MVSGCSIPSCIAPSGSTRSVTRRISGGISSGNYSCGP-VQHYAVRSCSIPYYSIQPLSCQPSCGISSCNYSCGPVEHCVVRPCPVPYYSI
Aca_EDSCP-C    --------------YSIQPLSCQPSCGISSCNYSCGP-IEHCVVRPCPVPYYSIQPQSCQPSCGFSPCNYPCGPTESCGVQSPSIQYYPI
Consensus      ........................s..........g.........scnepcv.qcpdS.v.i.p....vt.pGPils..pq...vgs...

Gga_BKfeather  AAVGNILSAEGVPISGGFGISGLGGRFSGR------------------------------------------------------RCLPY
Gga_BKclaw     PAIGSGMGGTFGRGAGFGGYGGLGG----YGGFYGLG----GYGGYGGFG--SCGYGG-WRRGLSYLSG---------------SCGPC
Gga_BKscale    PIFGGSSLGYGGSSLGYRGLYGYGGSSLGYGGLYGYGSSSLGYGGLYGYGGSSLGYGGLYGYGRSYGSGYCSPYSYRYNRYRRGSCGPC
Gga_EDbeta     PEVASGLSS--APAPGGLQVSGGARWARGYPVG---------------------------------------------------SCQPC
Aca_BK2        CGYGSCGYGGYGGYGGYGGYGGYGGYG-GYGG-WGYGGRGCGYGGWGGCGYGHCGYGG-WGNRCGYGGWGY------------------
Aca_BK28       CATGYGSCYSP------------------------------GYSGCYSPGYG-CNYGSGYGRKNCYGSSYPCSYPGYTRRCSYSSCGPC
Aca_BK7        CVGGYSGSYGSGYPGNFGGYLGSTGY-----------------------------------------------------------CRPC
Aca_EDSCP-N    QPLSCQPSCGI-SSCNYSC-GPVEHCVVRP------------------------------------------------------CPVPY
Aca_EDSCP-C    QSVSCQPSCGVQSSCAPSCYTPIQVQSCNP------------------------------------------------------CCSPC
Consensus      ...g...s.......g..g..g..g............................................................c.Pc
```

Suppl. Fig. S2. Chicken EDbeta and anole lizard EDSCP are related to beta-keratins. The amino acid sequences of 3 chicken beta-keratins (BK) and 3 lizard beta-keratins (BK) were aligned to those of chicken EDbeta and anole lizard EDSCP. The sequence of Aca_EDSCP was split into the N-terminal portion (Aca_EDSCP-N) and the C-terminal portion (Aca_EDSCP-C) to show sequence similarity (highlighted by yellow shading), which indicates an origin by sequence duplication within a single beta-keratin-like precursor gene. The N-terminus of Aca_EDSCP shows sequence similarity (identical residues shaded grey) to the N-terminus of Aca_BK7, i.e. the beta-protein encoded by the neighboring gene of Aca_EDSCP. The position of the beta-keratin signature motif is indicated by a pink box. Hash keys above the sequence alignment indicate the core-box (Alibardi et al, 2009), and asterisks indicate the extended region of sequence conservation. The accession numbers of Gga_EDbeta and Aca_EDSCP are shown in Suppl. Table S1. Aca_BK7 corresponds to the protein named Li_Ac_7 by Dalla Valle et al. (2010); coding sequence: NW_003338916.1, nucleotides 293755 - 294108). BKfeather corresponds to GGA_FK1 (Greenwold et al., 2010): NP_001264860, BKclaw corresponds to GGA_Claw1 (Greenwold et al., 2010): XP_001231443, BKscale corresponds to GGA_Scale1 (Greenwold et al., 2010): NP_001264908, Aca_BK2 corresponds to Li_Ac_2 (Dalla Valle et al., 2010): XP_003226366, and Aca_BK28 corresponds to Li_Ac_28 (Dalla Valle et al., 2010): XP_003226394. Aca, *Anolis carolinensis*; Gga, *Gallus gallus*.

```
 Hsa_LOR  MSYQKKQPTPQPPVD--------------CVKTSGGGG-------------------------------GGGGSGGGGCG
Gga_LOR1  MGSHQQKGEGQGISEQSGGCHGGGSGGSSCHGGGGGGSCHSSGG-------GGSIGY-QSQGSSC---HGGGSSGGGGAI
Gga_LOR2  MCS--RQSS--GGCHESSSQSGG------CCSGGSSSSYQAQGSSCC----GGSSGY-SM---------GGGYSGGSGGS
Gga_LOR3  MCS--RQSS--GGCHESSSQSGG------CCSGGSSSSYQAQGSSCC----GGSSGY-SM---------GGGYSGGSGGS
Aca_LOR1  MSGQQRQSS--GCCGRGGGGGGGGGGGSSCCGRRSGGSSSSSSSSCCCSSSRGSSGQ-QSQGSMCCGGGGGGGSGGGGSG
Aca_LOR2  MS-QQRQSGCCGSNRSPRCGRRGSGSGSSCCGGSSGGGSSCGRRGSGSSGCGGSSVGIAQVSGGSSCGRRGSGSSCCGGS

 Hsa_LOR  ---------------------------------------FFGGGGSGGGSSGSGC------------------------G
Gga_LOR1  YQTHISSSSFGGGGGG---------GGGSSGHQGQEPICIIGGGGGSSGGGGGSSHQSQGPICIGG-----------GGG
Gga_LOR2  SQKIIISSGGGGGGSSGCC------GGGSSSGGSSGGKIIIGGGGSSGGSSGCCSGGSSSYGMGGGYSS---------GG
Gga_LOR3  SQKIIISSGGGGGGSSGCC------GGGSSSGGSSGGKIIIGGGESSGGSSGCSSGGSSGYGIGGGYSS---------GG
Aca_LOR1  GQKIIIVSSGGSSGGGQCCCGGGGGGGGSSSGGGSGQIIISSGGGGSGGQSSGSCGGGSGGGSGGGMQQKIPCCGGGGGG
Aca_LOR2  S---VGIAQVSGGYGGSCC--------SSGGGSGQGVVIVSGGQGSSCCGSSGGIQQIRGSGCCSG-------------G

 Hsa_LOR  YSGGGGYSGGGCGGGSSGGGGGGGIGG-----------------CGGGSGGSVKYSGGGGSSG-----------------
Gga_LOR1  GGGGSDHQSQGPICIGGGGGGGGGGSGH--------QSKIPICISGGGGGK-GEGGSSHQGQGPICIGGGG--------G
Gga_LOR2  YSGSKSIIGGG-SSGGSSGCCGGGSSSGG-------SSGGKIIITGGGSSG-GSSGCCSGGSS---GYGIG--------G
Gga_LOR3  YSGSKSIIGGGGSSGGSSGCCGGGSSSGG-------SSGGKIIITGGGSSG-GSSGCCSGGSSYGGGYSSGSSGSKSIIG
Aca_LOR1  GSGGIKIIGGGSSGGGGGGGGGGGGGSGGMKSMGGGSSGGSSCGMGGGSGG-GGGSGGGGGSGGVKIIGGGSSGGGGGGG
Aca_LOR2  SSGGAVIIPAGVGGQSSGCCVGGGYGSGM------GQQKIPVIDSGFGSGGIGCGGGSGGGLGGQSIIVPVP-------G

 Hsa_LOR  -GGSG-------------------------------------CFSSGGGGSGCFSSGGGGSS------------------
Gga_LOR1  GGGGGGSSHQGQGP---------------------------ICIGGGGGGGG-----GGGSS---------HQGQGPICI
Gga_LOR2  GYSSGGYS----GSKS--------------------------IIGGGGSSGGSSGCCGGGSSSG------GSSGGKIIIT
Gga_LOR3  GGSSGGSS----GCCSGGSSGYGTSGYGSSRYGSGGSGQKIIISSGGGGGGGSSGCCGGGSSSG------GSSGGKIIIT
Aca_LOR1  GGGSGGMKSMGGGSSGGSSCGMGGGSGGGGGSGGGGGSGGIKIIGGGSSGGGGGGGGGGGGGSGGMKSMGGGSSGGSSCGM
Aca_LOR2  SGGSGG------------------------------------CYGGGQQVIGSGISSASGCCSG------DAGVGGVKVI

 Hsa_LOR  GGGSGCFSSGGGGSSGGGSGCFSSGGGGFSGQAVQCQSYGGVSSGGSSGGG-------------SGCFSSG---------
Gga_LOR1  GGGGGGKGEGGSSHQGQGPICIGGGGGGGGGGGSGHQSQGPICIGGGGGGGG--------GGGSSGHQGQGPICIGGGGG
Gga_LOR2  GGGSSGGSSGC---CSGGSSGYGIGGGYSSGGYSGSKSIIGG---GGSSGGSSG----CCGGGSSSGGSSGGKII---IT
Gga_LOR3  GGGSSGGSSGC---CSGGSSAYGIGGGYSSGGYSGSKSIIGG---GGSSGGSSG----CCGGGSSSGGSSGGKII---IT
Aca_LOR1  GGGSGGGGGSGGVKIIGGGSSSGGGGGGGGGGSGGMKSMGGGSSGGGSSGGSGGKTIIICGGSSGGGQSSGGQSSGGQSS
Aca_LOR2  GGGSGRVSPVCGGGVSGGGVKVIGGSGRVSPVCGGGVSGGGVKVIGGGSGRV--------SPVYGGGASCGGVKV---IG

 Hsa_LOR  GGGGSVCGYSGGGSGCGGGSSGGSGSGYVSSQQVTQTSCAPQPSYGGGSS------------------------------
Gga_LOR1  GGGGSGHQSQGPICIGGGSIGGGGGGGGSSHQGQGPICISGGGGGGGG--------------------------------
Gga_LOR2  GGGSSGG-SSG-CC-SGGSSGYGIGGGYSSGGYSGSKSIIGGGGSSGGSSG-----------------------------
Gga_LOR3  GGGSSGG-SSG-CC-SGGSSSYGIGGGYSSGGYSGSKSIIGGGGSSGGSSGFCGGGSSSGGYSEGKIIITGGGSSGGSSG
Aca_LOR1  GGQSSGGQSSG-CCMGGGSSGSGGGGGQTIIVPCGGSSGSGGQSSGGQSSSSCMGGGSSGGGGGGGGGGGGGGQNSGHQT
Aca_LOR2  GGSGRVSPVYGGGASCGGVKVIGGGSGRVSPVYGGGASCGGVKVIGGGSGR-----------------------------

 Hsa_LOR  -------------------------------GGGGSGG----------------------------------SGCFSSGG
Gga_LOR1  -----GGGSSHQSQG------------PICIGGGGGGG-----------GGGGSGHQSQGPICI--------GGGGGGGG
Gga_LOR2  C---CSGGSSGYGIGGGY-SSGGYSGSKSIIGGGSSGG----SSGCC-GGGSSSGGSSEGKIII--------TGGGSSGG
Gga_LOR3  C---CSGGSSGYGMGGGY-SSGGYSGSKSIIGGGSSGG----SSGCC-GGGSSSGGSSEGKIII--------TGGGSSGG
Aca_LOR1  IIVSCGGSSGGGGQSSRRRQSSGGQSSSSCMGGGSSGGGGGQSSGCCMGGGSGGGGGGGGQTIIVPCGGSSGSGGQSSGG
Aca_LOR2  VSPVYGGGVSGGGV--------------KVIGGGSGGG-----------GSICGGGVSGGGVKV--------IGGGSGGG

 Hsa_LOR  -------GGGSSGCGGGSSGIGSGCII---------SGGGSVCGGGSSG-------------------------------
Gga_LOR1  GGSGYQGQGPICIGGGGGGGGGSGHQSQGPICIGGGGGG----GGGGSGYQGQGPICIGGGGGGGSSHQSQGPICIGGG-
Gga_LOR2  -SSGCCSGGSSYGMGGGSSGGYSGSKSIIG--GGSSGGSSGCCGGGSSGYGSSGY----GSSSYGSGGSGQKIIISSGG-
Gga_LOR3  -SSGCCSGGSSYGMGGGSSGGYSGSKSIIG--GGSSGGSSGCCGGGSSGYGSSGY----GSSGYGSGGSGQKIIISSGG-
Aca_LOR1  QSSSCMGGGSSGGGGGGGGGQNSGHQTIIVSCGGSSGGGGQSSGGQSSGGQSSGCCMGGGSSGGGGGSGGQTIIVPCGGS
Aca_LOR2  ---GSICGGGLSDGGVKVVGGGSGRISPVYG-GGVSGGGVKVVGGGSGG----------GGSICGGGLSGGGVKVIGGG-

 Hsa_LOR  -------------------------GGGGGSS---------VGGSGSGKG------------------------------
Gga_LOR1  --GGGGGGGSGYQSQGPICIGGGGGGGGGGGSGHQGQGSICIIGGGSGGGGSSG-------------SGGMSMQQQTQPI
Gga_LOR2  -GGGGSSGCCGGGSSSGGSSGGKIIIGGGGSSGGSSGC--CGGGSSGGSGGSSG--HTIIISSGGGSGGYGQSSQQKCPI
Gga_LOR3  -GGGGSSGCCGGGSSSGGSSGGKIIVGGGGSSGGSSGC--CGGGSSGGSGGSSG--HTIIISSGGGSGGYGQSSQQKCPI
Aca_LOR1  SGGGGGGQSSGGQSSSCCMGGGSSSGGGGGGSGGQTIIIPCGGSSGGGGGGSSGGQSSGCCSGGGGGSSGGSSMQSKQSF
Aca_LOR2  --SGRVSPVCGGGS------GGQTIVVSGGSSGG------CCGGASSGTSVVVG---------------GGGSSQVKVPL

 Hsa_LOR  -VPICHQTQQKQAPTWPS----K
Gga_LOR1  --------------SWPPQTKHK
Gga_LOR2  VIPHIESHQTKQACYFPGQQ--K
Gga_LOR3  VIPHIESHQTKQACYFPGQQ--K
Aca_LOR1  SIPACLS-QTKQMNTWPSGQ--K
Aca_LOR2  VVPPCLG-QTKQVTSLPPSC--K
```

Suppl. Fig. S3. Amino acid sequence alignment of loricrin orthologs of human, chicken and lizard. Amino acid residues are highlighted by colors as in Figure 3. Hsa, *Homo sapiens*; Gga, *Gallus gallus*; Aca, *Anolis carolinensis*.

```
Aca_S100A11  MFKRSSRYTAGPSETERCIESLLAVFHKYAKGDRDANTLSKEEFKRFMNTELASLTKNQKDPAIVDRIGKKIDMNNDGNIDFEEFLNLVG
Hsa_S100A11  -----MAKISSPTETERCIESLIAVFQKYAGKDGYNYTLSKTEFLSFMNTELAAFTKNQKDPGVLDRMMKKLDTNSDGQLDFSEFLNLIG
Gga_S100A11  -----MSKVS-PTETERCIESLLAVFQRYAGREGDNLKLSKKEFRTFMNTELASFTKNQKDPAVVDRMMKRLDINSDGQLDFQEFLNLIG
 Hsa_S100A4  ----------MACPLEKALDVMVSTFHKYSGKEGDKFKLNKSELKELLTRELPSFLGKRTDEAAFQKLMSNLDSNRDNEVDFQEYCVFLS
 Gga_S100A4  ---------MMACPLEQALAVMVSTFHKYSGKEGDKFKLSKAELKELLSRELPAFGTKQMDEGEFRRLMNDLDHDKDSEVDFKEYACFLA
   Aca_EDKM  ----------MSRLIRAFTDMMEGNHKSNPKKVKEAETFKKSEFKKLIQQELSPV--QRSSSSKYKNMKNALDSDAELMTDKE---TVPC
   Gga_EDKM  ----------MSRLIKAITDMMDGS----ARRGKKSESFSRSEFKKLIQQEFAPV--KRSSTSKYHYIGSPLDSDTEPMNKKERGSSKTC
  Consensus  ..........m....e...d.$...f..ya.k......lsksEfk.l...Ela.....r.d..........lDs#.#...dfee......

Aca_S100A11  GIASACHSHVAASGKP-----
Hsa_S100A11  GLAMACHDSFLKAVPSQKRT-
Gga_S100A11  GIAVACHDALLVQPPHP----
 Hsa_S100A4  CIAMMCNEFFEGFPDKQPRKK
 Gga_S100A4  CVAMGYNEFFRDA--QRPRKK
   Aca_EDKM  AY-------------------
   Gga_EDKM  VY-------------------
  Consensus  ..a..................
```

Suppl. Fig. S4. Amino acid sequence alignment of EDKM and S100A proteins. The amino acid sequences of EDKM proteins were aligned to those of representative S100A proteins. The alignment was done with the Multalin algorithm using the following default settings. Symbol comparison table: BLOSUM62. Gap weight: 12. Gap length weight: 2. Consensus levels: high=90% (red letters), low=50% (blue letters). Consensus symbols: $ is anyone of LM; # is anyone of NDQEBZ. Aca, *Anolis carolinensis*; Gga, *Gallus gallus*; Hsa, *Homo sapiens*.

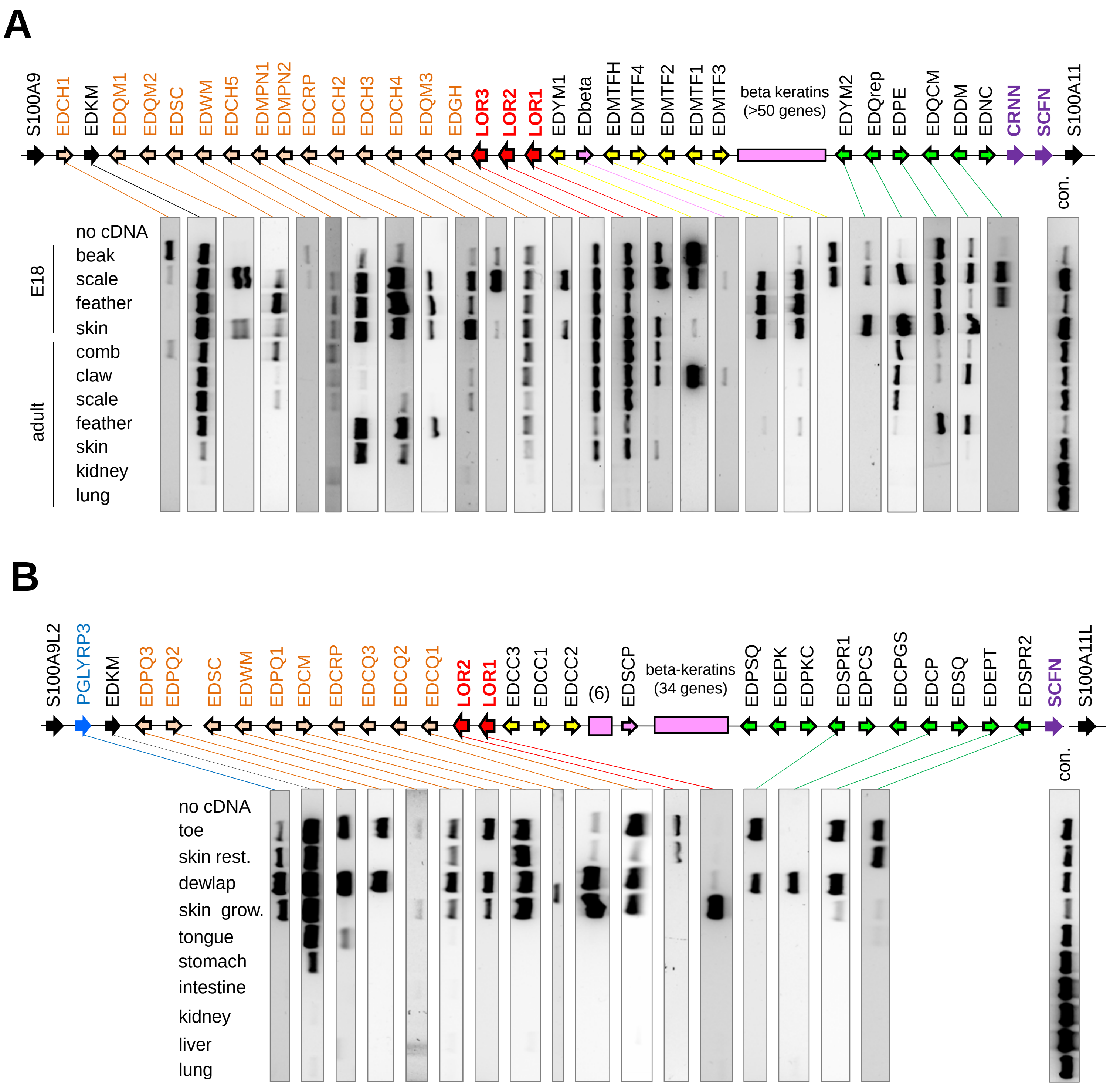

Suppl. Fig. S5. Comparison of expression patterns of EDC genes and their positions within the EDC. Images of RT-PCR results (Fig. 2A, B) were arranged along the EDC (Fig. 1) for (**A**) chicken and (**B**) lizard. Not for all genes the PCR amplification was done. skin rest., skin resting; skin grow., skin growing; con., control gene.

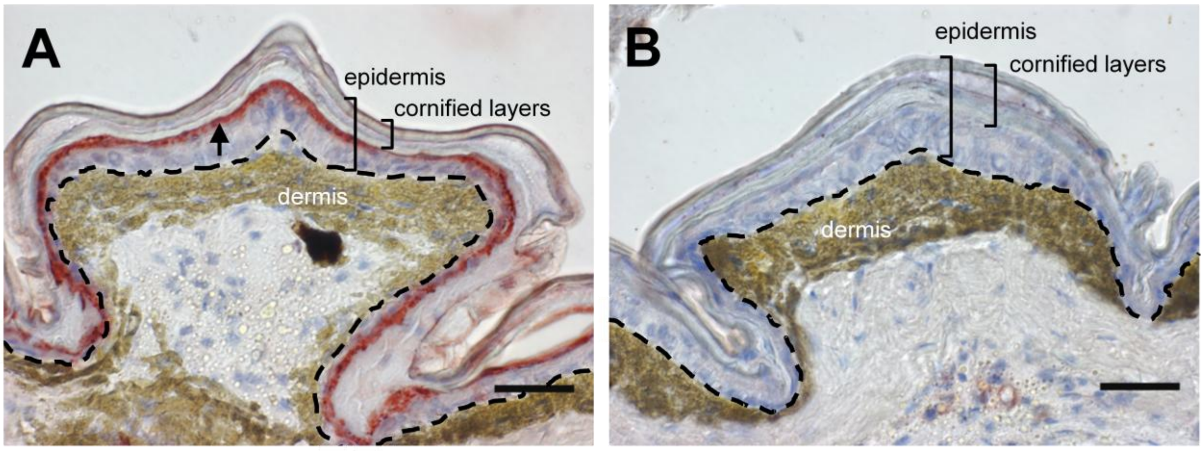

Suppl. Fig. S6. Immunohistochemical detection of loricrin 1 in the skin of the green anole lizard. Sections through the skin of *A. carolinensis* were immunostained with an antibody against loricrin 1 (red) (**A**). An arrow indicates the immunopositive layer of the epidermis. To confirm the specificity of the staining, the primary antibody was pre-incubated with a peptide corresponding to the epitope of the antibody, leading to the abrogation of the signal (**B**). Scale bar, 40 µm.

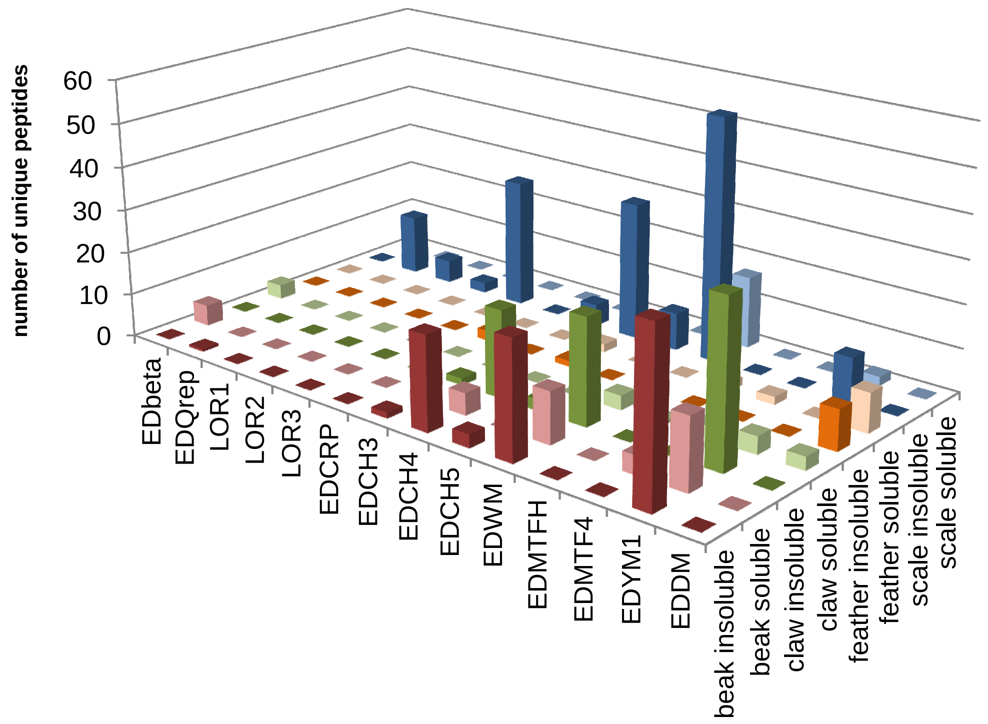

Suppl. Fig. S7. Proteomics of chicken cornification products. Beak (red), claw (green), feather (orange), and scale (blue) of chicken were analyzed by proteomics (Rice et al., 2013). The number of peptides corresponding to our 14 newly identified SEDC proteins are shown as bars (mean of preparations from 4 individual animals) devided into insoluble and soluble fractions. For details, see Suppl. Table S5.

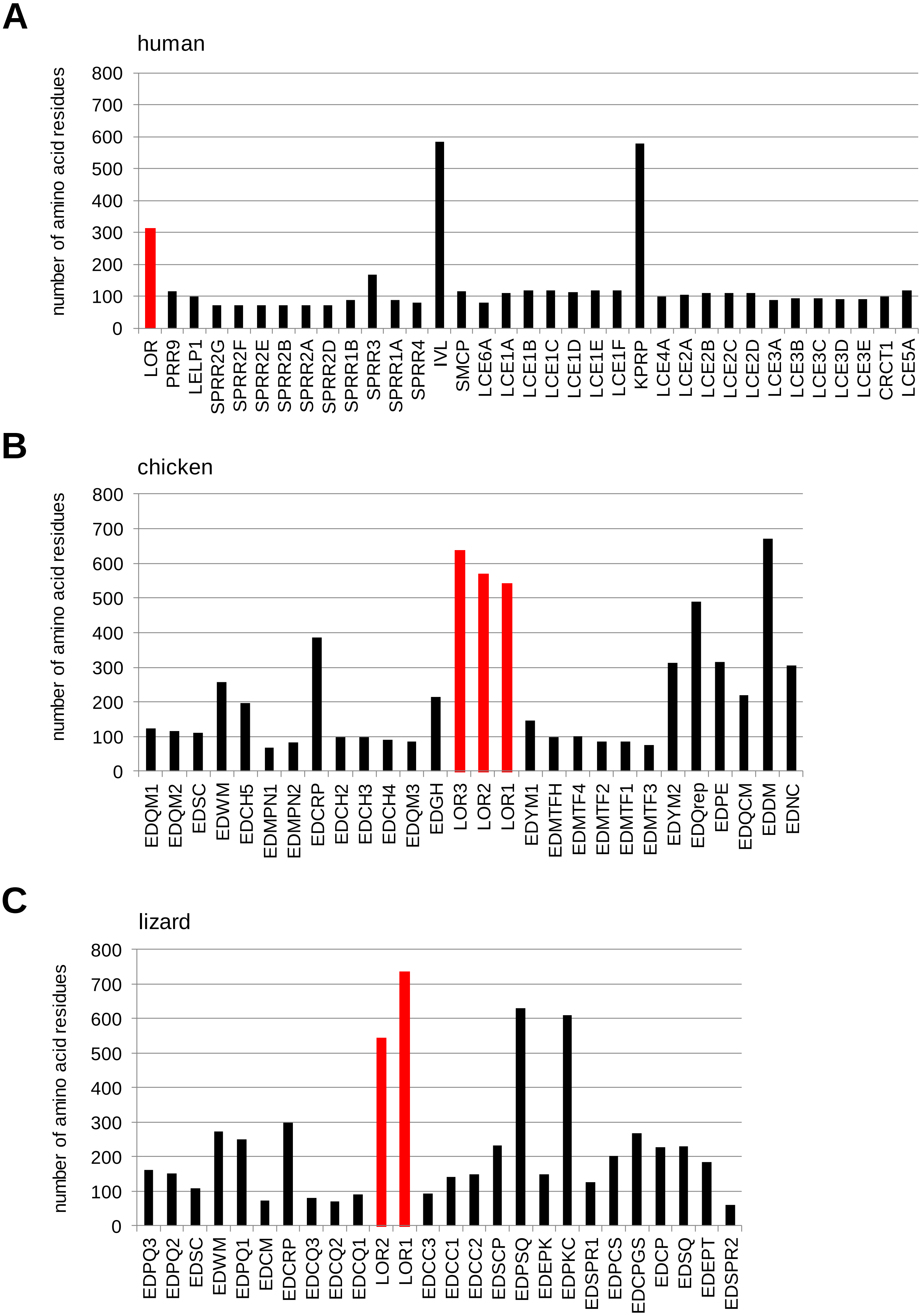

Suppl. Fig. S8. Lengths of SEDC proteins. The diagram shows the lengths of SEDC proteins measured as number of amino acid residues. The proteins are arranged in the order of the genes encoding them within the human (**A**), chicken (**B**) and lizard (**C**) EDC. Bars corresponding to loricrin orthologs are highlighted by red shading.

```
Hsa_Ivl    MSQQHTLPVTLSPALSQELLKTVPPPVNTHQEQMKQPTPLPPPCQKVPVELPVEVPSKQEEKHMTAVKGLPEQECEQQQKEPQEQELQQQHWEQHEEYQK
Gga_EDQrep MLCYQQ---QCFPPLHQEHIPIKCP--QTHPLMHSWQSELCTPQYVTPCVPRQRFLSSSFSPQQCVTQCIPRQQ--YATKCVQQQQCVTQHIPPARCVTT
Gga_Ivl    --------------------------------MHSWQSELCTPQYVTPCVPRQRFLSSSFSPQQCVTQCIPRQQ--YATKCVQQQQCVTQHIPPARCVTT
           *           * * **      *   **         *  *    *        *             * *      *  * *    **

Hsa_Ivl    AENPEQQLKQEKTQRDQQLNKQLEEEKKLLDQQLDQELVKRDEQLGMKKEQLLELPEQQEGHLKHLEQQEGQLKHPEQQEGQLELPEQQEGQLELPEQQE
Gga_EDQrep CVPQQSCAAQGMSQEPCVTKCMPQQQCATKCISQQQCATKCIPQQQCAARCVTTCIPQQPFLTKGIRQQHSATVCIPQHCVTTYAPHEQCATRCVTTC--
Gga_Ivl    CVPQQSCAAQGMSQEPCVTKCMPQQQCATKCISQQQCATKCIPQQQCAARCVTTCIPQQPFLTKGIRQQHSATVCIPQHCVTTYAPHEQCATRCVTTC--
                    *   *                     *   *   *             **    *   **        *          *

Hsa_Ivl    GQLELPEQQEGQLELPEQQEGQLELPEQQEGQLELPQQQEGQLELSEQQEGQLELSEQQEGQLKHLEHQEGQLEVPEEQMGQLKYLEQQEGQLKHLDQQE
Gga_EDQrep ----VPQQRATRCV---SQRYVTACAPQQCANKSIPQQQQCATKCIPQQQCATRCVTTCVPQ-------------PCETKGTSICVPQQQCATKCIPQQQ
Gga_Ivl    ----VPQQRATRCV---SQRYVTACAPQQCANKSIPQQQQCATKCIPQQQCATRCVTTCVPQ-------------PCETKGTSICVPQQQCATKCIPQQQ
                ** *           *        **      ****        **            *             * *  *      **    *   **

Hsa_Ivl    KQPELPEQQMGQLKHLEQQEGQPKHLEQQEGQLEQLEEQEGQLKHLEQQEGQLEHLEHQEGQLGLPEQQVLQLKQLEKQQGQPKHLEEEEGQLKHLVQQE
Gga_EDQrep CVTKCVPQQCAT-----------KCIPQQQCATKCIPQQQCATKCIPQQQCATKCIPQQQCAKCIPQQQCVT-KCIPQQQCVTKCIPQQQCVTKCAPQQ-
Gga_Ivl    CVTKCVPQQCAT-----------KCIPQQQCATKCIPQQQCATKCIPQQQCATKCIPQQQCAKCIPQQQCVT-KCIPQQQCVTKCIPQQQCVTKCAPQQ-
                  **               *   **         *    *   **          *      * **    *    **   *         *   **

Hsa_Ivl    GQLKHLVQQEGQLEQQERQVEHLEQQVGQLKHLEEQEGQLKHLEQQQGQLEVPEQQVGQPKNLEQEEKQLELPEQQEGQVKHLEKQEAQLELPEQQVGQP
Gga_EDQrep -------QCTKCIPQQQCATKCVPQQCAT-KCIPQQQCVTKCIPQQQCATKCAPQQCATKC----------IPQQQQCATKCVPQQCATKGIPQQHQCAT
Gga_Ivl    -------QCTKCIPQQQCATKCVPQQCAT-KCIPQQQCVTKCIPQQQCATKCAPQQCATKC----------IPQQQQCATKCVPQQCATKGIPQQHQCAT
                  *      **        **    *    *    *   ***       **                * **    *    * *    * *

Hsa_Ivl    KHLEQQEKHLEHPEQQDGQLKHLEQQEGQLKDLEQQKGQLEQPVFAPAPGQVQDIQPALPTKGEVLLPVEHQQQKQEVQWPPKHK
Gga_EDQrep KGILQQQQCVTKCVPQQSVTQCVPQQPYQ--------------------------------SGGVKISSHAKKYCSASKWP---W
Gga_Ivl    KGILQQQQCVTKCVPQQSVTQCVPQQPYQ--------------------------------SGGVKISSHAKKYCSASKWP---W
           *   **         *        **  *                                 * *              **
```

Suppl. Fig. S9. Amino acid sequence alignment of human involucrin (Ivl), chicken EDQrep and a previously predicted protein named chicken involucrin. Amino acid sequences of human involucrin, chicken EDQrep and chicken involucrin as predicted by Vanhoutteghem et al. (2008) were aligned. The positions of sequence identity are marked by asterisks. Amino acid residues involved in intermolecular crosslinking via transglutamination (Q, K) and disulfide bridge formation (C) are highlighted by color shading. Note that the previous chicken involucrin prediction represents the N-terminally truncated sequence of EDQrep. This discrepancy has been caused by the fact that the cDNA, from which the putative chicken involucrin was predicted, did not comprise the complete 5´-end of the open reading frame nor any sequence derived from the non-coding exon 1. Gga, *Gallus gallus*; Hsa, *Homo sapiens*.

A

>Gga_EDDM

```
MLCQTDCQRGLPCLPPHVVLVRNLP
VSRSVDPCNSVCSVSR
LNTCADPCYYARVPQGTTTYLKLGSCDL
RQIALDPCCLG
ITTLTDPCCQDVTRCS
STRCVDPCCCKVTECS
STRYVDSCCQDATQC
TTRCVDSCCQDATQC
TTRCVDPCCQDVTQC
TTRCVDPCRQDATQC
TTRCVDPCRQDATQC
TTRCVDPCCQDVTKC
TTRCVDPCRQDTTQC
TTRCVDPCCQDVTKC
TTRCIDPCRQDTTQC
TTRCVDPCCQDVTRC
TTRCVDPCCKEVTRC
TTRCVDPCCQDVTKC
TTRCVDPCCKEVTKCT
TTRCVDPCCKEVTRC
TTRCADPCCGEVTKC
TTKYVDPCCRPVTRC
ATTCVDPCCGRVTKC
TNKCVDPCYGAVTRC
STKCVEPCCEEVSKC
TSRCVDPCCREVTKC
TTRCVDPCCGRVTKC
-ARYKNPCCGEVSKC
TTKCVDPCCGRVTKY
----VDPCCREVTSC
KTRCVDPCCKEVTRC
TTTCVDPCCAEVTRC
ATKCIDPCCQDATKC
TTTCVDPCCREVTKC
TTTCVDPCCQEVTKC
TTTCVDPCCKEVTKC
TTTCVDPCCQEVTKC
TTTCVDPCCREVAKC
TTTCVDPCCQDATQC
TTRCADPCCQDVTQC
TSTCVDPCVPPCFVRP
TPLCASICGRHY
SISCADICCRK
```

B

>Gga_LOR1

```
MGSHQQKGEGQGISEQSGGCHGGGSGGSS
CHGGGGGGSCHSSGGGGS
IGYQSQGSSCHGGGSSGGGG
AIYQTHISSSSFGGGGGGGGGS
SGHQGQEPICIIGGGGGSSGGGGG
SSHQSQGPICIGGGGGGGGG
SDHQSQGPICIGGGGGGGGG
SGHQSKIPICISGGGGGKGEGG
SSHQGQGPICIGGGGGGGGGGG
SSHQGQGPICIGGGGGGGGGGG
SSHQGQGPICIGGGGGGKGEGG
SSHQGQGPICIGGGGGGGGGGG
SGHQSQGPICIGGGGGGGGGGGS
SGHQGQGPICIGGGGGGGGG
SGHQSQGPICIGGGSIGGGGGGGG
SSHQGQGPICISGGGGGGGGGGG
SSHQSQGPICIGGGGGGGGGGG
SGHQSQGPICIGGGGGGGGGG
SGYQGQGPICIGGGGGGGGG
SGHQSQGPICIGGGGGGGGGG
SGYQGQGPICIGGGGGGG
SSHQSQGPICIGGGGGGGGGG
SGYQSQGPICIGGGGGGGGGGG
SGHQGQGSICIIGGGSGGGGSSGSGG
MSMQQQTQPISWPPQTKHK
```

C

>Aca_EDSC

```
MSQQVQRGSSCCCSA
CCGGSRSGG
CCSSSRSSGG
CCSGRRSSGG
CCGSSRSSGG
CCGSSSGGKYVQQAQASA
GCGSSSGG
CCGGSSSSG
CCSGGLSQQKQVKMCPQQLK
```

Suppl. Fig. S10. Examples of repetitive sequence elements in SEDC proteins of sauropsids. The amino acid sequences of chicken EDDM (**A**), chicken loricrin 1 (**B**) and anole lizard EDSC (**C**) were arranged to reveal sequence repeats. Abundant amino acid residues are color-coded as shown in Figure 3. Aca, *Anolis carolinensis*; Gga, *Gallus gallus*.

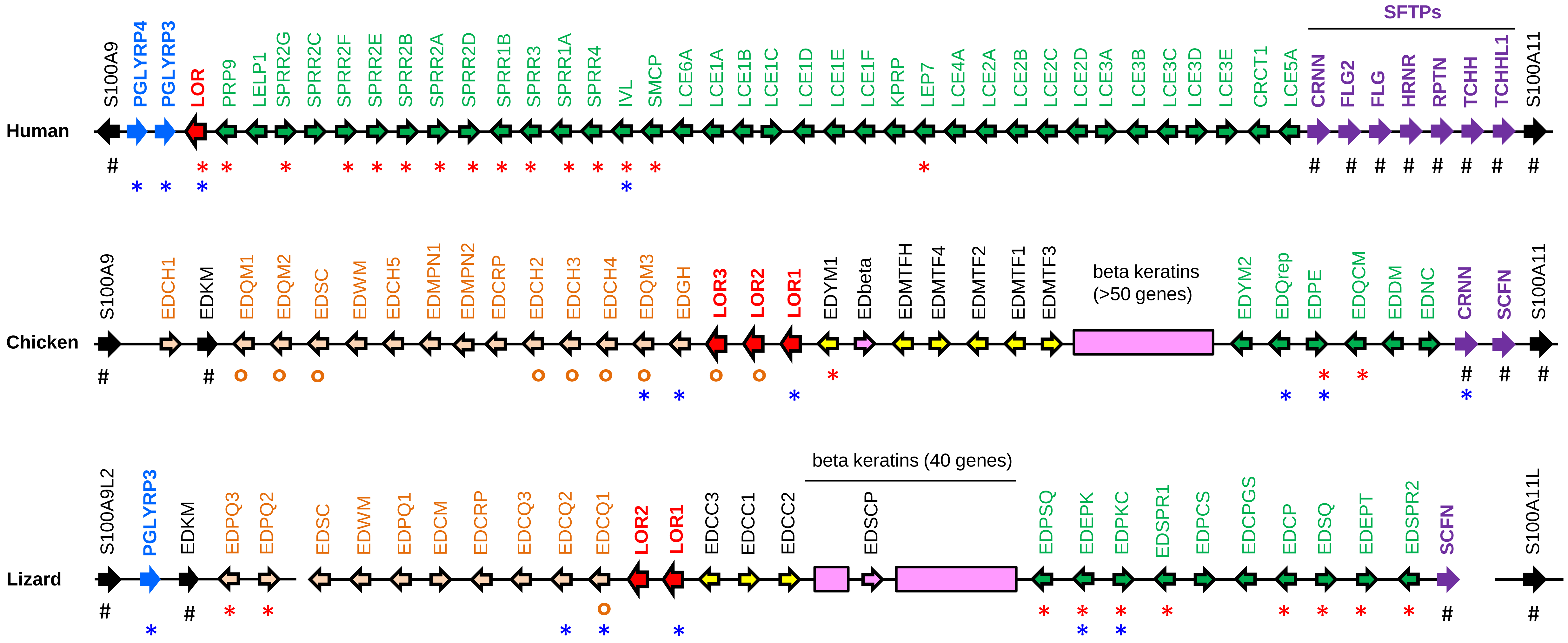

Suppl. Fig. S11. Distribution of conserved sequence motifs among genes of the epidermal differentiation complex (EDC) in amniotes. The hash keys mark genes encoding proteins with an S100 domain or S100 domain-like sequences (EDKM). Red and blue asterisks mark genes that encode proteins with the conserved N- and C-terminal sequence motifs, respectively (Fig. 4). Orange circles mark genes that encode proteins with the sauropsidian N-terminal sequence motif shown in Suppl. Fig. S12.

```
Gga EDCH2    MCSRGSS--CH
Gga EDCH3    MCSRGSS--CH
Gga EDCH4    MCSRGSS--CH
Gga LOR2     MCSRQSSGGCH
Gga LOR3     MCSRQSSGGCH
Gga EDSC     MCSRQDKDQCH
Gga EDQM1    MCSRQDRDQCH
Gga EDQM2    MCSRQDRDQCH
Gga EDQM3    MCSRADRDQCH
Aca EDCQ1    MCSRGDRD-CH

Consensus    MCSRxxxxxxCH
```

Suppl. Fig. S12. Identification of an amino acid sequence motif that is conserved at the N-terminus of a group of sauropsidian SEDC proteins. Amino acid sequences of the N-terminus of SEDC proteins were aligned. Amino acid residues present at conserved positions are highlighted by colors. Aca, *Anolis carolinensis*; Gga, *Gallus gallus*.

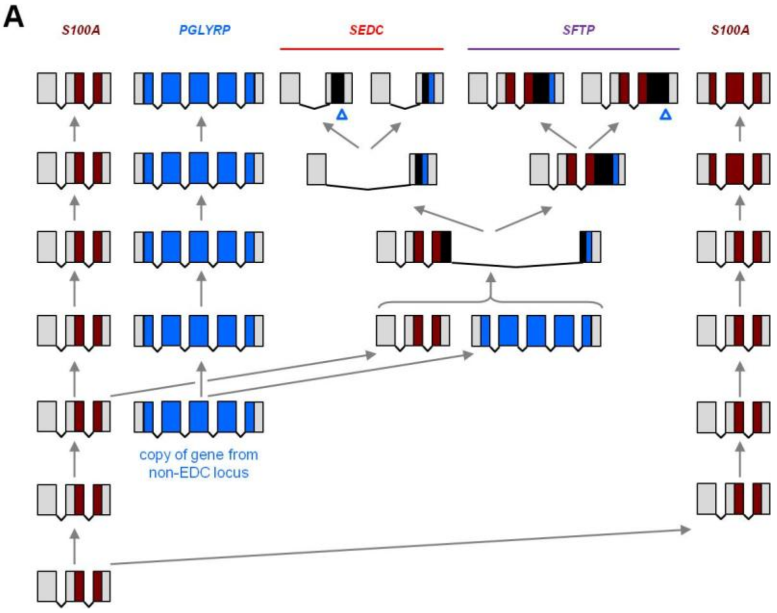

<u>Scenario 1:</u> The fusion of an S100A gene and a PGLYRP gene yielded a common ancestor of SEDC and SFTP genes that encoded a protein containing the C-terminal sequence motif. The PGLYRP-derived C-terminal motif was lost in subsets of SEDCs and SFTPs.

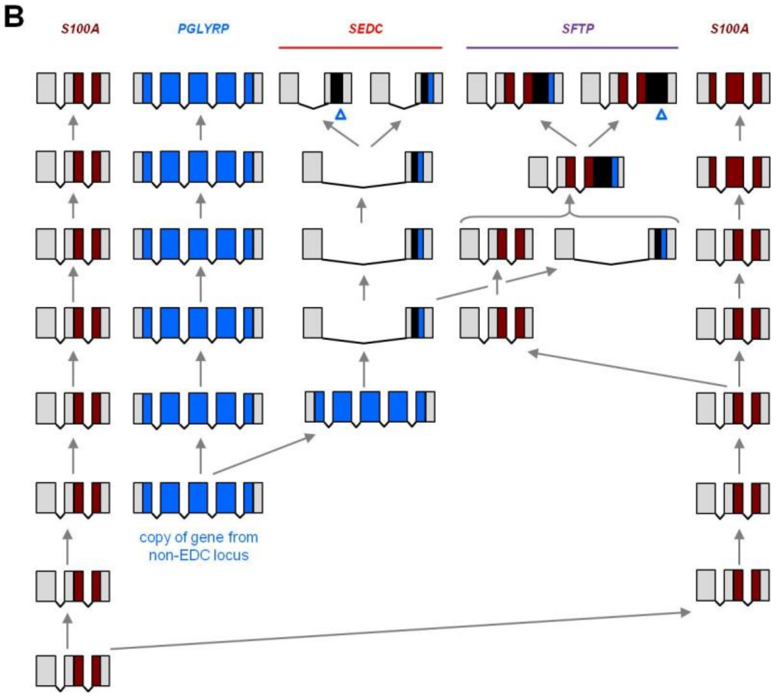

<u>Scenario 2:</u> The mutation of a PGLYRP gene (loss of exons and new start of coding sequence) yielded an SEDC gene. Fusion of this SEDC gene to an S100A gene yielded an SFTP gene. The PGLYRP-derived C-terminal motif was lost in subsets of SEDCs and SFTPs.

Suppl. Fig. S13. Scenarios for the origin of SEDC and SFTP genes (continued on the next page).

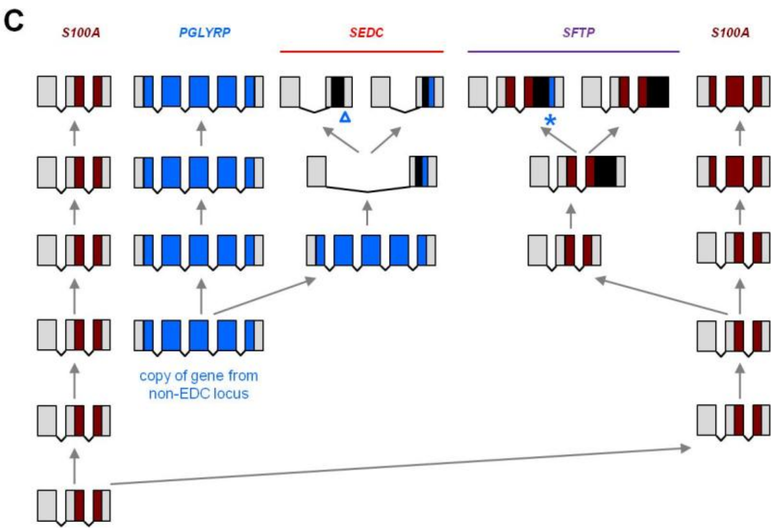

Scenario 3: The mutation of a PGLYRP gene yielded an SEDC gene. The extension of the coding sequence of an S100A gene yielded an SFTP. The C-terminal motif was lost in subsets of SEDCs and SFTPs. Independently, a similar C-terminal sequence motif originated in a subset of SFTPs.

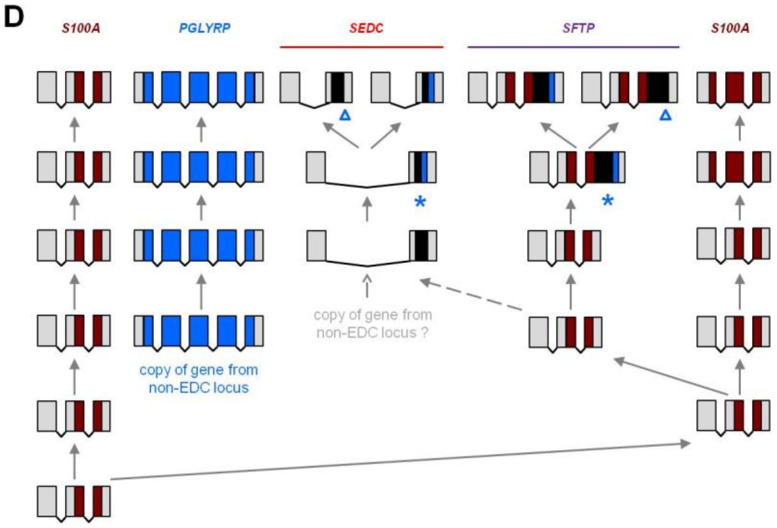

Scenario 4: The extension of an S100A coding sequence yielded an SFTP. The ancestral SEDC originated by mutation of an S100A gene or as a copy of a gene from another locus. The C-terminal sequence motif originated independently from PLGLYRP in ancestral SEDC and ancestral SFTP. The C-terminal motif was lost in subsets of SEDCs and SFTPs.

Suppl. Fig. S13 (continued). Scenarios for the origin of SEDC and SFTP genes. Four alternative scenarios for the origin of SEDC and SFTP genes during the evolution of the EDC are schematically depicted. Exons are indicated by boxes, in which the non-coding regions are shaded grey and the coding regions are shaded in colors or in black. Identical colors indicate common ancestry and black indicates newly originated coding sequences. All genes shown are transcribed from left to right. Asterisks indicate an origin of the C-terminal sequence motif (Figure 4) independently from PGLYRP. Loss of the motif is indicated by the symbol Δ. Note that the evolution of EDKM was not integrated into these schemes. In most S100A genes, exons 2 and 3 contain the entire coding sequence whereas in S100A11 (located next to SFTPs) the open reading frame starts in exon 1. In scenario 4 the insertion of the PGLYRP gene into the EDC locus may have occurred after the origin of the primordial SEDC and SFTP genes but prior to divergence of sauropsids and mammals.
